# Machine learning-based prediction of recurrent extrahepatic bile duct stones after common bile duct exploration: a comparative study of models and SHAP-driven interpretability analysis

**DOI:** 10.3389/fmed.2025.1691519

**Published:** 2025-12-12

**Authors:** Yugang Cao, Xun Hu, Jun Guo, Tao Fang

**Affiliations:** Department of Hepatobiliary Surgery, Huangshi Central Hospital, Hubei Key Laboratory for Kidney Disease Pathogenesis and Intervention, Hubei Polytechnic University School of Medicine, Huangshi, Hubei, China

**Keywords:** machine learning, recurrent extrahepatic bile duct stones, common bile duct exploration, SHAP-driven interpretability analysis, key risk factors

## Abstract

**Purpose:**

This study aimed to construct and compare machine learning models for predicting recurrent extrahepatic bile duct stones after common bile duct exploration and to clarify the contribution of key risk factors using SHAP analysis, thereby providing a reliable tool for clinical risk assessment and intervention.

**Methods:**

Retrospective analysis of 1,363 patients (2010–2024, Huangshi Central Hospital/Honghu People’s Hospital) with extrahepatic bile duct stones (156 recurrent cases). LASSO regression selected 8 predictors; 9 machine learning models were built, evaluated by AUC, accuracy, etc., and SHAP interpreted the optimal model.

**Results:**

Random Forest (RF) performed best: training/validation/external cohort AUC 97.99%/93.66%/83.1%, accuracy 0.953/0.902/0.829. SHAP identified maximum stone diameter, common bile duct diameter, and direct bilirubin as top risks, with nonlinearity (stones >15 mm elevated risk) and synergistic interactions.

**Conclusion:**

Random Forest (RF) is confirmed as the most reliable tool for predicting recurrent extrahepatic bile duct stones post-common bile duct exploration, outperforming other models in generalization. SHAP analysis clarifies that max stone diameter, CBD diameter, and direct bilirubin (with nonlinear effects like stones >15 mm elevating risk) are key synergistic risks. This study enables personalized clinical risk assessment and targeted interventions to reduce postoperative recurrence.

## 1 Introduction

Extrahepatic bile duct stones are a common hepatobiliary disorder, with common bile duct exploration as the primary treatment. However, postoperative stone recurrence remains a critical issue, occurring in 4–30% of cases, which impairs patient quality of life, increases healthcare burdens, and raises risks of acute cholangitis, biliary pancreatitis, and cholangiocarcinoma (1, 2).

Traditional risk factors (e.g., intrahepatic bile duct stones, large common bile duct diameter, previous biliary surgery) (3, 4) have limited predictive accuracy, as recurrence depends on complex interactions between clinical, laboratory, and imaging variables. In recent years, machine learning has shown promise in hepatobiliary disease prediction—for instance, Zhang et al. (5) developed models for hepatolithiasis recurrence with AUC > 0.9, and Chen et al. (6) improved choledocholithiasis prediction via machine learning to optimize ERCP decisions. Zhao et al. (7) also applied machine learning to predict biliary surgery complications, demonstrating its superiority over traditional statistics.

Notably, few studies focus on machine learning-based prediction of extrahepatic bile duct stone recurrence after common bile duct exploration. Existing models lack comprehensive variable selection or validation, and their poor interpretability hinders clinical adoption. The SHAP method addresses this by quantifying feature contributions: Wang et al. (8) used SHAP to interpret hilar cholangiocarcinoma recurrence models, aiding clinical understanding of risk drivers.

This study aims to construct and compare machine learning models for predicting extrahepatic bile duct stone recurrence post-exploration, and to use SHAP to interpret the optimal model. The goal is to identify key risk factors and their interactions, providing a reliable tool for personalized risk assessment and targeted prevention.

## 2 Materials and methods

### 2.1 Study subjects

This study adopted a retrospective design to enroll patients with extrahepatic bile duct stones. The training cohort and validation cohort data were both derived from patients diagnosed with extrahepatic bile duct stones and treated at Huangshi Central Hospital (including its main campus and Huang jinshan Campus) and Honghu People’s Hospital between October 2010 and October 2024. The research team conducted a retrospective review of the medical records of these patients, and all cases of extrahepatic bile duct stones (including recurrent cases) were confirmed through clinical manifestations (such as abdominal pain, jaundice, or cholangitis) and imaging examinations, including abdominal ultrasound, magnetic resonance cholangiopancreatography (MRCP), endoscopic retrograde cholangiopancreatography (ERCP), or computed tomography (CT).

To ensure the homogeneity and representativeness of the study population, the following inclusion criteria were strictly implemented: (1) age ≥ 18 years (no upper age limit specified, consistent with the baseline data range reflecting clinical practical enrollment); (2) clear diagnosis of extrahepatic bile duct stones via imaging and clinical evaluation; (3) history of common bile duct exploration as the primary treatment for extrahepatic bile duct stones (CBDE was defined as open or laparoscopic surgical exploration of the common bile duct with stone extraction); (4) complete follow-up data were available to confirm recurrence of gallstones (recurrence was defined as the reappearance of extrahepatic bile duct stones at least 3 months after initial surgical choledocholithotomy).

Exclusion criteria were established to reduce confounding factors and ensure data quality, including: (1) patients with incomplete medical records (missing key variables such as laboratory indicators like direct bilirubin, ALP, GGT, or imaging parameters like maximum stone diameter and common bile duct diameter); (2) patients with a follow-up duration of less than 3 months (insufficient to assess stone recurrence); (3) patients with concurrent severe diseases that could interfere with the judgment of bile duct stone recurrence or survival status, such as advanced cholangiocarcinoma, severe liver failure, or other end-stage organ diseases; (4) receipt of alternative primary treatments for extrahepatic bile duct stones—including endoscopic retrograde cholangiopancreatography (ERCP) with endoscopic sphincterotomy (EST) alone (without CBDE), percutaneous transhepatic cholangioscopy (PTCS), or medical dissolution therapy (e.g., ursodeoxycholic acid monotherapy for stone dissolution).

After applying the above inclusion and exclusion criteria, a total of 1,480 initially screened patients with extrahepatic bile duct stones were filtered, and 1,363 patients were finally enrolled in the study. The missing rates of all key variables (including laboratory indicators, imaging parameters, comorbidity information, and follow-up data) involved in the study were systematically counted, and the specific results are presented in Table S1 in Supplementary Materials. Overall, the missing rates of all variables were below 5%, with the highest missing rate observed for “follow-up duration (≥3 months)” (3.85%) and “maximum stone diameter” (2.8%), the missing rates of core predictors (e.g., direct bilirubin: 1.9%, common bile duct diameter: 2.6%) were far below the 10% threshold that may introduce significant selection bias. Before excluding patients with incomplete records, we first cross-checked electronic and paper-based medical records and consulted attending physicians or contacted patients/family members to supplement missing data. For patients with unresolved key variable missingness after supplementation (*n* = 117, accounting for 7.9% of the initial cohort), we excluded them to avoid imputation bias—sensitivity analysis confirmed no significant differences in baseline characteristics between excluded and included patients (all *p* > 0.05), indicating minimal impact on cohort representativeness.

**Figure 1:**
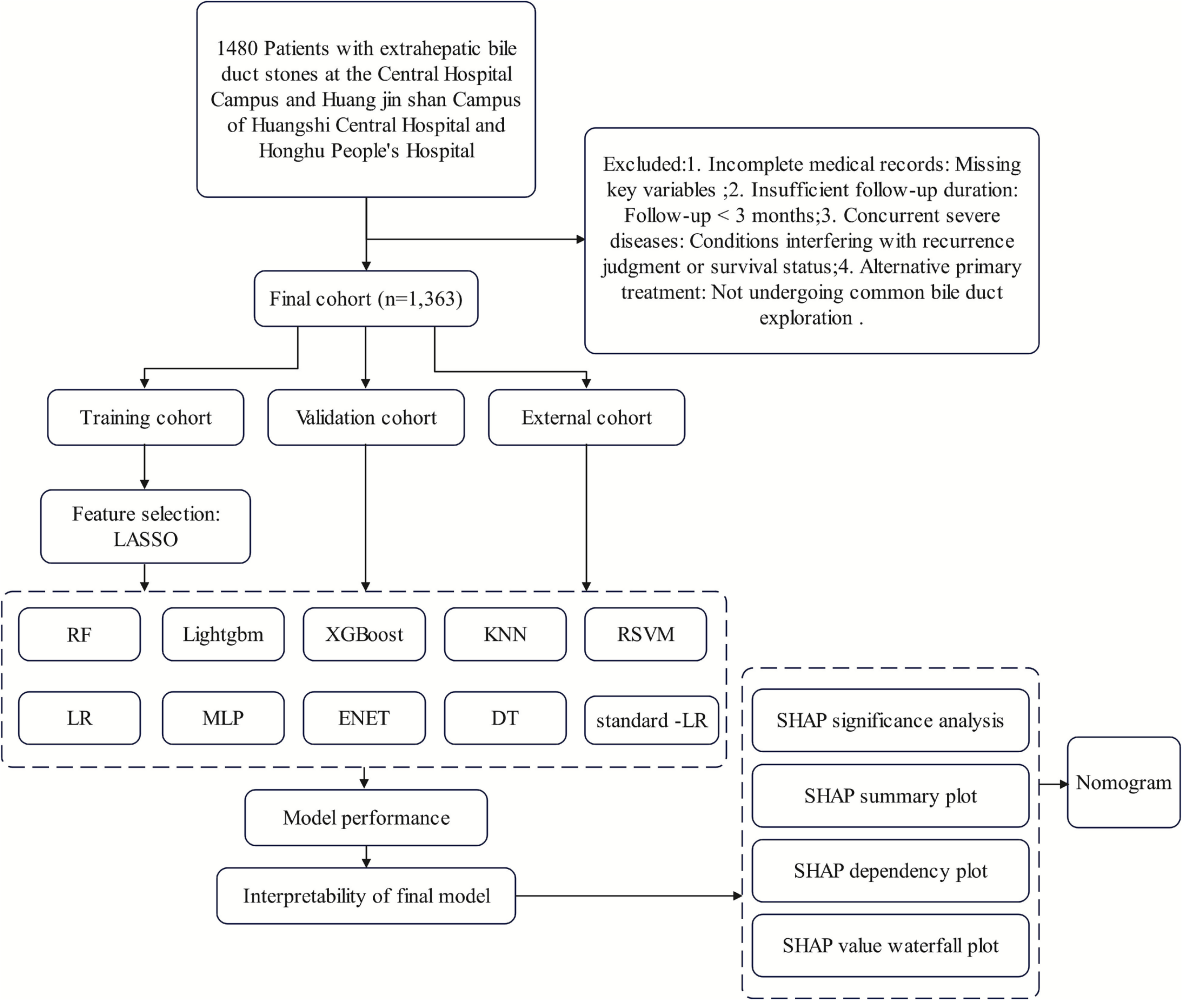
Flowchart outlining patient enrollment and study design.

Using R software (version 4.5.1) with a random number generator seed of 1,234, the 1,258 patients were randomly divided into a training cohort and a validation cohort at a ratio of 7:3. Among them, 881 patients constituted the training cohort (used for variable selection and model development), and the remaining 377 patients formed the validation cohort (used for evaluating the generalization performance of the developed models). Additionally, an external cohort of 105 patients was included to further assess model performance. The patient enrollment process is illustrated in Figure 1, which presents a detailed flowchart outlining the steps of cohort screening, application of inclusion/exclusion criteria, and division into the training and validation cohorts.

### 2.2 Data collection

#### 2.2.1 Recurrent extrahepatic bile duct stones

During data collection, recurrent extrahepatic bile duct stones were defined as stones reappearing in the extrahepatic bile ducts (including the common bile duct and hepatic duct) after patients underwent common bile duct exploration (CBDE) for stone removal or dissolution, with recurrence confirmed at least 3 months post-primary treatment (following the previous surgery for recurrent extrahepatic bile duct stones, imaging confirmed no residual stones in the left or right hepatic ducts or extrahepatic bile ducts). All cases were diagnosed through clinical manifestations such as abdominal pain, jaundice, or cholangitis, with imaging studies including abdominal ultrasound, magnetic resonance cholangiopancreatography (MRCP), or endoscopic retrograde cholangiopancreatography (ERCP) used to determine stone quantity, size, and location. Asymptomatic recurrent stones—identified through follow-up imaging without clinical symptoms—were also included in the analysis. Additionally, stone characteristics (size, quantity), associated clinical symptoms, and re-treatment plans were documented to ensure data completeness and accuracy.

#### 2.2.2 Laboratory and imaging findings

Laboratory findings included measurements of serum biochemical indicators and hematological parameters. Serum biochemical indicators comprised direct bilirubin, alkaline phosphatase (ALP), gamma-glutamyl transferase (GGT), alanine transaminase (ALT), and aspartate transaminase (AST), while hematological parameters included white blood cell count (WBC), C-reactive protein (CRP), prothrombin time (PT), and activated partial thromboplastin time (APTT). All laboratory tests were performed using standard clinical procedures in the hospital’s central laboratory, with results recorded as continuous variables.

Imaging findings were primarily derived from abdominal ultrasound, magnetic resonance cholangiopancreatography (MRCP), endoscopic retrograde cholangiopancreatography (ERCP), computed tomography (CT), or magnetic resonance imaging (MRI). Key imaging parameters included maximum stone diameter (measured as the largest dimension of detected stones), common bile duct diameter (measured at the midpoint of the common bile duct), and the presence of concurrent intrahepatic bile duct stones (recorded as binary: “yes” or “no”). For patients with recurrent extrahepatic bile duct stones, imaging was also used to confirm stone location, number, and associated complications (e.g., biliary obstruction). All imaging data were reviewed and interpreted by two radiologists specializing in abdominal imaging, with discrepancies resolved through consensus.

#### 2.2.3 Health status

Health status assessment included the collection of basic demographic information, lifestyle factors, and comorbidities.

##### 2.2.3.1 Lifestyle factors

Smoking history and alcohol consumption were recorded as binary variables (“yes” or “no”). Smoking history was defined as having a history of regular smoking for at least 6 months; alcohol consumption was defined as consuming alcohol at least once a week for more than 3 months.

##### 2.2.3.2 Comorbidities

The presence of chronic diseases was confirmed based on medical records and diagnostic criteria, including.

##### 2.2.3.3 Hypertension

Defined as a history of hypertension diagnosed by a physician, use of antihypertensive medications, or systolic blood pressure ≥140 mmHg and/or diastolic blood pressure ≥90 mmHg on at least two measurements.

##### 2.2.3.4 Diabetes mellitus

Defined as a history of diabetes diagnosed by a physician, use of antidiabetic medications (oral hypoglycemic agents or insulin), or fasting blood glucose ≥7.0 mmol/L or random blood glucose ≥11.1 mmol/L.

##### 2.2.3.5 Hyperlipidemia

Defined as a history of hyperlipidemia diagnosed by a physician, use of lipid-lowering medications, total cholesterol ≥6.2 mmol/L, triglycerides ≥2.3 mmol/L, low-density lipoprotein cholesterol ≥4.1 mmol/L, or high-density lipoprotein cholesterol <1.0 mmol/L.

##### 2.2.3.6 Chronic liver disease

Including chronic hepatitis (viral hepatitis B, hepatitis C, etc.), cirrhosis, non-alcoholic fatty liver disease, etc., confirmed by clinical diagnosis, laboratory tests, or imaging findings.

##### 2.2.3.7 Previous surgical history

Information on previous biliary surgery was collected (binary: “yes” or “no”), including procedures such as cholecystectomy, bile duct exploration, or endoscopic sphincterotomy.

##### 2.2.3.8 Clinical symptoms

The presence of pain and fever was recorded during the study period. Pain was defined as abdominal pain (localized or diffuse) reported by the patient; fever was defined as an axillary temperature ≥37 °C. Both were recorded as binary variables (“yes” or “no”).

All health status data were extracted from electronic medical records, and missing information was supplemented by reviewing paper medical records or consulting the attending physicians to ensure accuracy and completeness.

##### 2.2.3.9 Previous surgical history

Information on previous biliary surgery (PBS) was collected as a binary variable (“yes” or “no”). PBS was defined as any prior surgical procedure involving the biliary tract performed ≥ 3 months before the index common bile duct exploration (CBDE), including but not limited to cholecystectomy (laparoscopic or open), open/ laparoscopic common bile duct exploration, bile duct reconstruction, and endoscopic retrograde cholangiopancreatography (ERCP) with biliary stenting (excluding diagnostic ERCP without interventional manipulation). Procedures unrelated to the biliary system (e.g., abdominal hernia repair, gastrointestinal surgery) were not classified as PBS.

### 2.3 Follow-up

Follow-up was initiated on the first day after patients’ discharge from the hospital post-common bile duct exploration (CBDE) and terminated upon confirmation of extrahepatic bile duct stone (EHBDS) recurrence (≥3 months post-surgery, verified by MRCP/ERCP/CT), patient death from non-stone-related causes, study conclusion (October 2024), or patient refusal to continue follow-up.

Patients were stratified into high-, medium-, and low-risk groups based on key predictors identified by the Random Forest model (maximum stone diameter, common bile duct diameter, direct bilirubin, concurrent intrahepatic bile duct stones, previous biliary surgery):

High-risk: Follow-up every 3 months (within 1 year post-surgery) and every 6 months (after 1 year);
Medium-risk: Follow-up every 6 months (within 1 year) and every 12 months (after 1 year);
Low-risk: Follow-up every 12 months (within 1 year) and every 18 months (after 1 year).

Follow-up methods included routine telephone/online questionnaire surveys (assessing symptoms like abdominal pain, jaundice, and fever) and annual on-site visits (for physical examination, laboratory tests including direct bilirubin, ALP, GGT, and imaging exams such as abdominal ultrasound/MRCP). Emergency follow-up was triggered for suspected recurrence symptoms, with patients instructed to seek hospital care within 24 h.

Data were recorded via an electronic data capture system, with 10% of records quality-checked monthly. Patients provided informed consent prior to follow-up, and the privacy of their data was ensured through encrypted storage. Detailed follow-up procedures are available in the Supplementary Materials.

### 2.4 Statistical analysis

In this study, continuous variables are presented as median with mean ± standard deviation unless otherwise specified, and group differences are identified using univariate analysis. Categorical variables are reported as frequencies and proportions for each patient group, and compared using chi-square tests or Fisher’s exact tests as appropriate. All statistical analyses were performed using R software (version 4.5.1), and a two-sided *p*-value < 0.05 was considered statistically significant.

Using R’s built-in random number generator with a seed value of 1,234, the 1,258 cases collected from two campuses of Huangshi Central Hospital were randomly split in a 7:3 ratio into a training set (*n* = 881) and a validation set (*n* = 377); additionally, 105 cases from Honghu People’s Hospital were designated as the external validation set. All patients in the training set (from Huangshi Central Hospital) were included in variable selection and model development. To reduce potential multicollinearity and prevent overfitting, we applied L1-penalized least absolute shrinkage and selection operator (LASSO) regression with 10-fold cross-validation strictly constrained within the training cohort (*n* = 881, from Huangshi Central Hospital). The tuning parameter lambda (*λ*) was determined exclusively by the performance of training folds (using the lambda.1se criterion), and data from both the validation set (*n* = 377, from Huangshi Central Hospital) and the external validation set (*n* = 105, from Honghu People’s Hospital) were not accessed during the variable selection process—this ensured that feature selection (ultimately retaining 8 variables with significant predictive power) was free from data leakage and only reflected patterns inherent in the training data. LASSO achieves feature selection by shrinking variable coefficients via a penalty term, where the magnitude of each coefficient is regulated by the tuning parameter lambda.

Subsequently, predictive models were developed using nine machine learning algorithms: logistic regression (LR), decision tree (DT), elastic net (ENet), extreme gradient boosting (XGBoost), k-nearest neighbors (KNN), light gradient boosting machine (LightGBM), multilayer perceptron (MLP), regularized support vector machine (RSVM), and Random Forest (RF). To benchmark the performance of these machine learning models against traditional statistical methods, we additionally constructed a standard logistic regression (standard-LR) model as a reference. The standard-LR model used the same eight key predictors selected by LASSO regression (to ensure fair comparison), with fixed parameters: binomial distribution for binary classification (recurrence vs. non-recurrence) and logit link function, without regularization (to represent the most commonly used traditional logistic regression approach in clinical research). Unlike the regularized LR (included in the nine machine learning models), which applies penalty terms to coefficients, standard-LR retains the original linear regression framework to avoid over-optimization and align with real-world clinical statistical practice.

Machine learning methods can handle a large number of predictors, rely on fewer model assumptions, and do not require pre-specifying model terms, enabling flexible, data-driven detection of interaction effects. Notably, for the Random Forest (RF) model—later identified as the optimal model—we implemented class weighting during training to avoid bias from unequal class distribution (11.6% recurrence rate in the full cohort): Class weights were assigned inversely proportional to class frequencies in the training set [weight for recurrence class = total training samples/(2 × number of training recurrence samples); weight for non-recurrence class = total training samples/(2 × number of training non-recurrence samples)]. This adjustment increased the model’s cost of misclassifying the minority “recurrence” class, improving detection of high-risk patients without overfitting.

For all machine learning models and the standard-LR model, stability was ensured through five-fold cross-validation (note: standard-LR, as a linear model with no tunable hyperparameters, only underwent five-fold cross-validation for performance verification without grid search). Model performance was evaluated using the same set of metrics: area under the receiver operating characteristic curve (AUC), accuracy, specificity, sensitivity, recall, and F-measure. The optimal model was selected based on the highest AUC and accuracy values from the validation set. Calibration curves were then used to assess the agreement between predicted and observed recurrent extrahepatic bile duct stone incidence in the training and validation sets, while decision curve analysis (DCA) was employed to evaluate net clinical benefit—with direct comparisons between the top-performing machine learning model (RF) and standard-LR to quantify improvements in clinical utility.

We applied the SHapley Additive exPlanations (SHAP) method to interpret the optimal machine learning model (RF). Rooted in game theory, SHAP values quantify the contribution of each feature to individual predictions, effectively explaining feature impact for each case. Feature importance was assessed using SHAP summary plots and SHAP feature importance rankings. We further explored feature effects on outcome prediction through SHAP dependence plots and used SHAP waterfall plots to clarify how individual features influenced predictions for specific patients. No SHAP analysis was performed for standard-LR, as its linear nature allows direct interpretation of coefficients (though limited by its inability to capture non-linear and interaction effects), which is consistent with traditional statistical model interpretation practices.

To further validate the robustness of the eight core predictive variables identified via L1-penalized LASSO regression and to address two critical biases inherent in retrospective studies of postoperative recurrence—immortal-time bias (arising from misclassifying the “risk-free interval” between surgery and follow-up initiation) and informative censoring bias (resulting from censoring events correlated with recurrence likelihood)—a LASSO-COX proportional hazards regression model was additionally constructed and detailed in Supplementary Material 4. This model was developed strictly using data from the training cohort (*n* = 881, from two campuses of Huangshi Central Hospital) to avoid data leakage, with 10-fold cross-validation employed to optimize the penalty parameter lambda (*λ*, determined via the lambda.1se criterion) and ensure consistency with the earlier variable selection framework. To mitigate biases, the LASSO-COX model incorporated time-dependent covariates (e.g., dynamic changes in direct bilirubin levels within one month postoperatively) to address immortal-time bias and applied inverse probability of censoring weighting (IPCW) to adjust for informative censoring. Supplementary Material 4 provides comprehensive documentation of the model’s construction process, including detailed parameter settings, bias adjustment workflows, and performance validation results (e.g., C-index for discriminative ability, calibration curves for agreement between predicted and observed recurrence probabilities). This supplementary analysis not only corroborates the predictive value of the eight core variables but also enhances the methodological rigor of the study’s statistical framework by accounting for potential biases unaddressed in the primary ML models.

## 3 Results

### 3.1 Baseline characteristics

This study included a total of 1,480 patients from the medical records system of Huangshi Central Hospital and Honghu People’s Hospital. After screening, a total of 1,363 patients were enrolled, among which 156 cases were recurrent extrahepatic bile duct stones after choledochotomy and met the selection criteria. As shown in Table 1, there were significant differences between the biliary stone recurrence group and non-biliary stone recurrence group in terms of age, maximum stone diameter (MSD), common bile duct diameter (CBDD), direct bilirubin, ALP and GGT. The distribution of variables between the training (*n* = 881), validation (*n* = 377), and external (*n* = 105) cohorts was well balanced, with no notable differences observed (*p* > 0.05) (see Table 1).

**Table 1:** Comparison of characteristics between the biliary stone recurrence group and non-biliary stone recurrence group, as well as between training and validation cohorts.

Variable	Training cohorts (*n* = 881)	Validation cohorts (*n* = 377)	External cohort (*n* = 105)	Statistic	*P*	Non-biliary stone recurrence group (*n* = 1,207)	Biliary stone recurrence group (*n* = 156)	Statistic	*P*
Age	57.6(15.1)	56.6(14.4)	60.7(15.3)	H = 5.78	0.0555	56.8(15.0)	63.7(12.9)	W = 67,506	<0.001
BMI	24.1(3.47)	24.2(3.39)	24.5(3.74)	H = 1.49	0.474	24.1(3.47)	24.6(3.50)	W = 86,687	0.107
Maximum stone diameter	9.47(5.84)	9.85(6.34)	9.80(5.56)	H = 2.58	0.276	8.89(5.63)	15.1(5.55)	W = 38,984	<0.001
Common bile duct diameter	12.8(5.83)	12.9(5.85)	12.7(5.12)	H = 1.72	0423	12.3 (5.59)	17.0 (5.53)	W = 50,090	<0.001
Operation duration	78.8(22.3)	78.5(21.5)	79.1(24.6)	H = 1.37	0.504	79.1 (22.6)	76.0 (19.4)	W = 104,637	0.0233
Postoperative hospital stay	6.96(2.03)	6.98(1.99)	6.89(2.23)	H = 0.0488	0.976	6.99 (2.03)	6.88 (2.03)	W = 97,074	0.527
Direct bilirubin	26.8(41.5)	26.1(37.4)	46.7(30.7)	H = 77.1	<0.001	24.7 (37.4)	55.3 (48.0)	W = 52,560	<0.001
ALP	103(25.9)	102(26.4)	105(27.7)	H = 0.921	0.631	101 (25.5)	119 (25.7)	W = 57,290	<0.001
GGT	49.1(24.7)	49.7(26.2)	49.2(25.5)	H = 0.0954	0.953	47.9 (24.5)	60.1 (27.1)	W = 67,608	<0.001
ALT	31.3(17.3)	31.1(9.72)	33.6(16.3)	H = 5.4	0.0673	30.9 (13.3)	35.5 (26.9)	W = 84,871	0.045
AST	36.2(12.8)	35.5(11.9)	34.2(14.4)	H = 1.93	0.382	35.7 (12.2)	37.4 (15.9)	W = 90,906	0.484
WBC	7.24(2.12)	7.08(2.10)	7.36(1.96)	H = 2.33	0.312	7.18 (2.10)	7.44 (2.09)	W = 86,750	0.11
CRP	7.26(1.96)	7.29(2.01)	7.36(1.97)	H = 0.73	0.694	7.27 (1.96)	7.31 (2.10)	W = 94,564	0.928
PT	12.0(0.952)	12.0(0.893)	12.0(0.942)	H = 0.142	0.932	12.0 (0.932)	12.1 (0.966)	W = 90,008	0.371
APTT	35.0(2.96)	34.9(3.17)	34.8(2.44)	H = 0.34	0.259	35.0 (2.98)	34.7 (2.95)	W = 96,889	0.553
Gender									
Female	429(48.7%)	194(51.5%)	54 (51.4%)	χ2 = 0.948	0.623	603 (50.0%)	74 (47.4%)	χ2 = 0.258	0.611
Male	452(51.3%)	183(48.5%)	51(48.6%)			604 (50.0%)	82 (52.6%)		
Smoking									
No	496(56.3%)	219(58.1%)	59(56.2%)	χ2 = 0.361	0.835	682 (56.5%)	92 (59.0%)	χ2 = 0.25	0.617
Yes	385(43.7%)	158(41.9%)	46(43.8%)			525 (43.5%)	64 (41.0%)		
Drinking									
No	443(50.3%)	212(56.2%)	51(48.6%)	χ2 = 4.22	0.121	635 (52.6%)	71 (45.5%)	χ2 = 2.51	0.113
Yes	438(49.7%)	165(43.8%)	54(51.4%)			572 (47.4%)	85 (54.5%)		
Hypertension									
No	789(89.6%)	323(85.7%)	91(86.7%)	χ2 = 4.12	0.128	1,071 (88.7%)	132 (84.6%)	χ2 = 1.88	0.17
Yes	92(10.4%)	54(14.3%)	14(13.3%)			136 (11.3%)	24 (15.4%)		
Diabetes									
No	764(86.7%)	339(89.9%)	91(86.7%)	χ2 = 2.58	0.275	1,056 (87.5%)	138 (88.5%)	χ2 = 0.0473	0.828
Yes	117(13.3%)	38(10.1%)	14(13.3%)			137(12.3%)	18(12.3%)		
Hyperlipidemia									
No	772(87.6%)	340(90.2%)	91(86.7%)	χ2 = 1.95	0.378	1,071 (88.7%)	132 (84.6%)	χ2 = 1.88	0.17
Yes	109(12.4%)	37(9.8%)	14(13.3%)			136 (11.3%)	24 (15.4%)		
Chronic liver disease									
No	756(85.8%)	324(85.9%)	85(81.0%)	χ2 = 1.88	0.391	1,029 (85.3%)	136 (87.2%)	χ2 = 0.272	0.602
Yes	125(14.2%)	53(14.1%)	20(19.0%)			178 (14.7%)	20 (12.8%)		
Concurrent intrahepatic bile duct stones									
No	716(81.3%)	314(83.3%)	84(80.0%)	χ2 = 0.948	0.622	1,040 (86.2%)	74 (47.4%)	χ2 = 136	<0.001
Yes	165(18.7%)	63(16.7%)	21(20.0%)			167 (13.8%)	82 (52.6%)		
Pain									
No	428(48.6%)	195(51.7%)	51(48.6%)	χ2 = 1.08	0.583	604 (50.0%)	70 (44.9%)	χ2 = 1.28	0258
Yes	453(51.4%)	182(48.3%)	54(51.4%)			603 (50.0%)	86 (55.1%)		
Fever									
No	496(56.3%)	190(50.4%)	59(56.2%)	χ2 = 3.82	0.148	662 (54.8%)	83 (53.2%)	χ2 = 0.0913	0.763
Yes	385(43.7%)	187(49.6%)	46(43.8%)			545 (45.2%)	73 (46.8%)		
Previous biliary surgery									
No	756(85.8%)	332(88.1%)	90(85.7%)	χ2 = 1.19	0.551	1,071 (88.7%)	107 (68.6%)	χ2 = 46.1	<0.001
Yes	125(14.2%)	45(11.9%)	15(14.3%)			136 (11.3%)	49 (31.4%)		
Intraoperative T tube placement									
No	777(88.2%)	335(88.9%)	91(86.7%)	χ2 = 0392	0.822	1,065 (88.2%)	138 (88.5%)	χ2 = <0.001	1
Yes	104(11.8%)	42(11.1%)	14(13.3%)			142 (11.8%)	18 (11.5%)		
Recurrent biliary lithiasis									
No	783(88.9%)	329(87.3%)	95(90.5%)	χ2 = 1.09	0.58				
Yes	98(11.1%)	48(12.7%)	10(9.5%)						

Data are presented as *n* (%) or mean (standard deviation).

To confirm the homogeneity of the study cohort (pure CBDE post-surgical patients), we further verified the primary treatment modality of all 1,363 enrolled patients via operative notes and procedure records. Among them, 100% (*n* = 1,363) underwent CBDE as the sole primary treatment: 21.8% (*n* = 297) received open CBDE, and 78.2% (*n* = 1,066) received laparoscopic CBDE. No patients with primary ERCP/EST, PTCS, or medical dissolution therapy were included, confirming that the cohort was free of mixed treatment pathways. This homogeneity ensures that the recurrence mechanisms analyzed (e.g., residual stone fragments, biliary stasis post-surgical ductal changes) are specific to CBDE, enhancing the external validity of our findings for CBDE post patients.

### 3.2 Spearman correlation analysis of continuous variables

**Figure 2:**
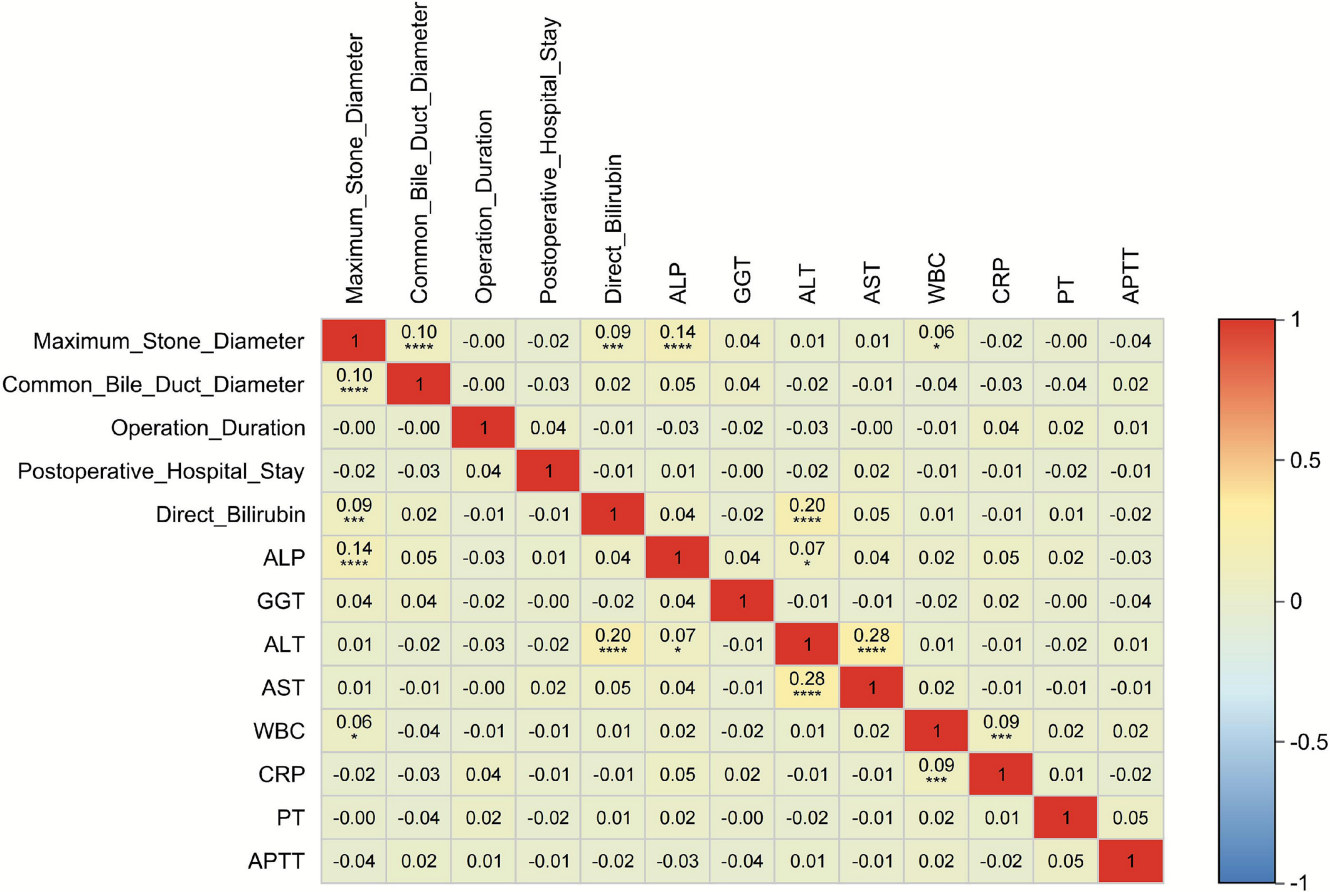
Heatmap of Continuous Variable Correlations.

To explore potential associations among key continuous variables in this study—including maximum stone diameter, common bile duct diameter, operation duration, postoperative hospital stay, direct bilirubin, ALP, GGT, ALT, AST, WBC, CRP, PT, and APTT—we performed Spearman rank correlation analysis in the training cohort to quantify pairwise correlations, with results presented in Figure 2. The analysis revealed that most correlation coefficients were small in magnitude (|r| ≤ 0.28), with only a few reaching moderate levels. The strongest correlation was observed between ALT and AST (r = 0.28), followed by the association between direct bilirubin and ALT (r = 0.20), both indicating weak-to-moderate positive relationships. Additionally, ALP showed a weak positive correlation with maximum stone diameter (r = 0.14), and WBC exhibited a weak positive correlation with CRP (r = 0.09). The absolute values of most other correlations ranged between 0.01 and 0.10, suggesting no meaningful linear relationships among the variables.

These findings indicate that the continuous variables included in this study are largely independent in terms of biological and clinical characteristics, with no evidence of strong collinearity. This supports the validity of subsequent multivariable modeling approaches—such as LASSO regression and machine learning algorithms—by confirming minimal interference among predictors, thereby enabling more stable estimation of each variable’s independent effect on outcomes (e.g., risk of recurrent extrahepatic bile duct stones) and reducing the risk of estimation bias due to multicollinearity.

### 3.3 Variable selection

This study was a retrospective analysis using existing clinical data. As no formal sample size calculation was performed *a priori*, we aimed to include the largest possible cohort from the database, ultimately enrolling 1,363 patients (stratified into a training cohort of 881, a validation cohort of 377, and an external cohort of 105). Initial analysis incorporated 28 variables (Table 1). Given the binary outcome of interest (recurrence vs. non-recurrence, 156 recurrent events in total), the final cohort met the widely accepted “10 events per variable (EPV)” rule (156 events/8 predictors ≈ 20 EPV), ensuring robustness of the results.

**Figure 3:**
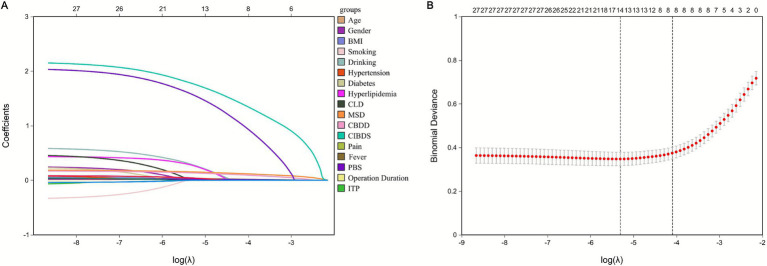
Presentation of the results of the LASSO regression analysis. **(A)** LASSO regression model screening variable trajectories; **(B)** LASSO Regression Model Factor Selection: the left dashed line represents the optimal lambda value (lambda·min), while the right dashed line marks the lambda value within one standard error of the optimal (lambda.1se).

To reduce potential multicollinearity and prevent overfitting, we applied the Least Absolute Shrinkage and Selection Operator (LASSO) regression with L1 penalty, strictly combined with 10-fold cross-validation within the training cohort (*n* = 881) (validation and external cohort data were not accessed during this step to avoid data leakage). LASSO performs feature selection by penalizing the regression coefficients, effectively shrinking their absolute values—a process controlled by the tuning parameter lambda. Through this dimensionality reduction, eight key predictors with significant predictive power were selected based on the lambda.1se criterion (Figures 3A,B). The final set of selected features included: age, maximum stone diameter, common bile duct diameter, concurrent intrahepatic bile duct stones, previous biliary surgery, direct bilirubin, ALP, and GGT (the coefficients are 0.02, 0.12, 0.135, 0.096, 1.383, 0.995, 0.012, 0.016 in order).

We then applied nine machine learning algorithms—Random Forest (RF), Light Gradient Boosting Machine (LightGBM), Extreme Gradient Boosting (XGBoost), K-Nearest Neighbors (KNN), Regularized Support Vector Machine (RSVM), Logistic Regression (Logistic), Multilayer Perceptron (MLP), Elastic Net (ENet), and Decision Tree (DT)—to predict the primary outcome exclusively from the training dataset (*n* = 881). The modeling process incorporated five iterations of 5-fold cross-validation along with grid search for hyperparameter tuning (detailed in Supplementary Material 3), ensuring strong generalization while minimizing overfitting.

### 3.4 Model performance and comparisons

**Figure 4:**
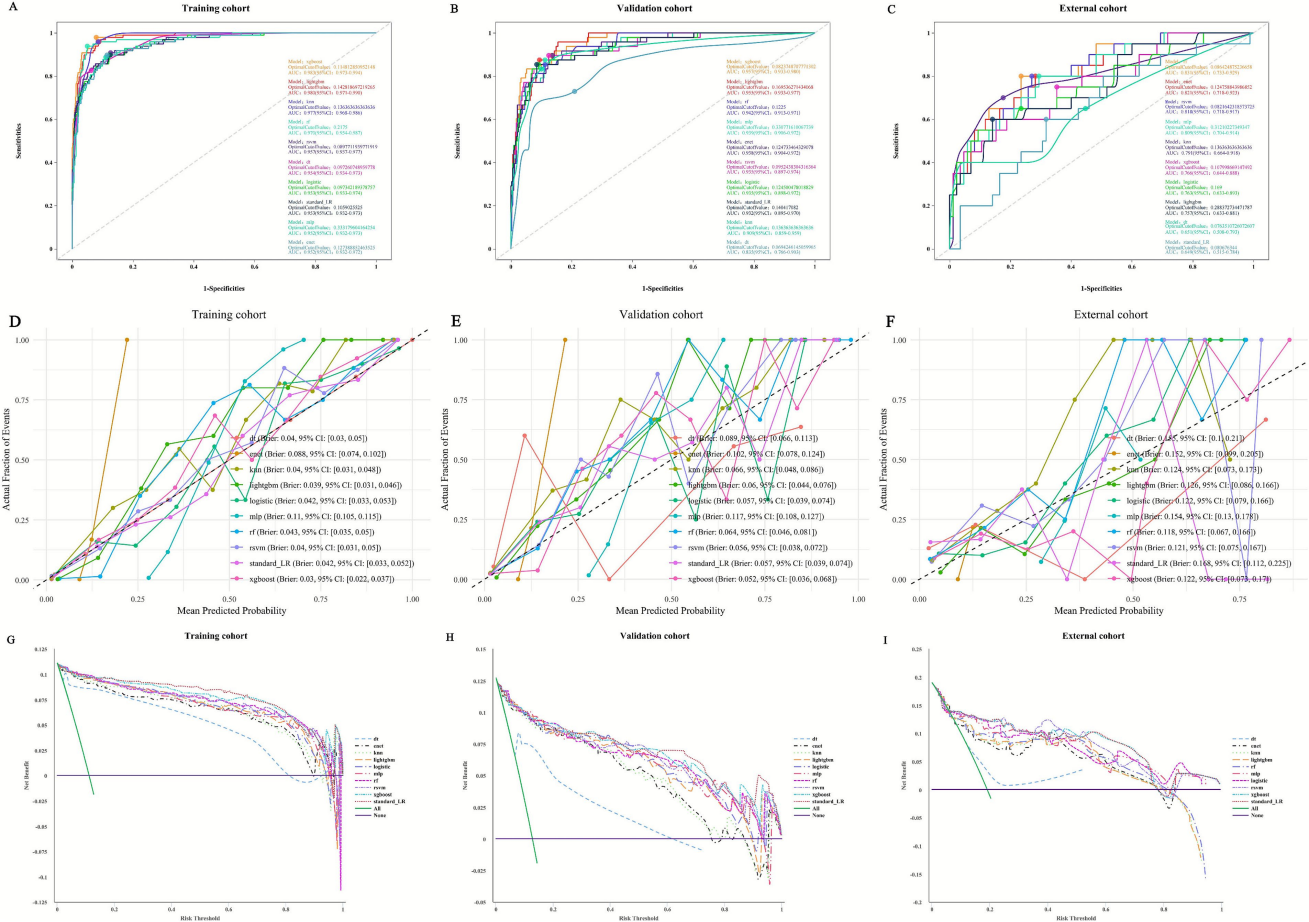
Comprehensive evaluation of machine learning and standard-LR models. **(A)** ROC curves and AUC values of the training cohort. **(B)** ROC curves and AUC values of the validation cohort. **(C)** ROC curves and AUC values of the external cohort. **(D)** Calibration curves of machine learning models in the training cohort, **(E)** validation cohort, and **(F)** external cohort. **(G)** Training cohort DCA curve, **(H)** validation cohort DCA curve, and **(I)** external cohort DCA curve.

To comprehensively evaluate the performance of machine learning models in predicting recurrent extrahepatic bile duct stones after common bile duct exploration, nine machine learning algorithms—Logistic Regression (Logistic), Elastic Net (ENet), Decision Tree (DT), Random Forest (RF), Extreme Gradient Boosting (XGBoost), Regularized Support Vector Machine (RSVM), Multilayer Perceptron (MLP), Light Gradient Boosting Machine (LightGBM), and K-Nearest Neighbors (KNN)—were developed, with traditional standard logistic regression (standard-LR) as a reference. Model performance was assessed using core metrics including accuracy, sensitivity (sens), specificity (spec), F-measure (f_meas), and area under the receiver operating characteristic curve (ROC-AUC), and supplemented by calibration curves and decision curve analysis (DCA) (Figure 4) to fully reflect discriminative ability, calibration degree, and clinical practical value. Comparisons were conducted in the training cohort (*n* = 881), validation cohort (*n* = 377), and external cohort (*n* = 105) to identify the optimal model and verify its generalization performance across different populations.

In the training cohort, all nine machine learning models demonstrated predictive capability, but the Random Forest (RF) model stood out with the best comprehensive performance—this superiority was also visually confirmed by Figure 4. As shown in Figure 4A (ROC curves of the training cohort), the RF model’s ROC curve was the closest to the upper-left corner of the coordinate system, corresponding to its ROC-AUC of 0.97, which was higher than most other models (e.g., logistic: 0.935, ENet: 0.938, DT: 0.835). In terms of quantitative metrics, RF achieved the highest accuracy (0.953), indicating the highest overall correctness of predictions; a sensitivity of 0.98, enabling effective identification of patients at high risk of recurrence and minimizing missed diagnoses; a specificity of 0.95, ensuring accurate recognition of non-recurrent patients to avoid unnecessary clinical interventions; and the highest F-measure (0.824), reflecting superior balance in identifying positive (recurrent) and negative (non-recurrent) samples. From the perspective of calibration (Figure 4D, calibration curves of the training cohort), the RF model had a low Brier score of 0.043, and its predicted recurrence probability was highly consistent with the actual observed recurrence rate—its calibration curve was the closest to the “perfectly calibrated” diagonal line, indicating that the model’s output probability could reliably reflect the true risk of recurrence. DCA (Figure 4G, training cohort DCA curve) further confirmed rf’s clinical value: across all risk thresholds, its curve remained above those of other models, meaning that using RF for risk prediction could minimize unnecessary interventions for low-risk patients and missed diagnoses for high-risk patients, achieving the highest net clinical benefit. Other models showed clear limitations: the Decision Tree (DT) model had an accuracy (0.95) close to that of RF but a significantly lower sensitivity (0.796) and F-measure (0.78) (see Table 2), and its ROC curve in Figure 4A was noticeably lower than RF’s, alongside a low ROC-AUC (0.835), indicating weak recognition of recurrent cases. The Extreme Gradient Boosting (XGBoost) model matched rf’s sensitivity (0.98) but lagged in accuracy (0.927), specificity (0.921), and F-measure (0.75) (see Table 2), and its DCA curve in Figure 4G was below rf’s, reflecting lower net clinical benefit. The Light Gradient Boosting Machine (LightGBM) had a slightly higher ROC-AUC (0.955) than RF but lower accuracy (0.914), specificity (0.907), and F-measure (0.714) (see Table 2). Regularized Support Vector Machine (RSVM), Multilayer Perceptron (MLP), Logistic Regression (Logistic), and Elastic Net (ENet) models exhibited moderate performance, with accuracy ranging from 0.877 to 0.897, sensitivity from 0.878 to 0.908, and F-measure from 0.622 to 0.654 (see Table 2) their ROC curves in Figure 4A were all below rf’s, and their calibration curves in Figure 4C showed greater deviations from the perfect calibration line. The K-Nearest Neighbors (KNN) model had moderate accuracy (0.919) and sensitivity (0.959) but a lower F-measure (0.726) and lacked stability, as evidenced by its relatively fluctuating calibration curve in Figure 4C.

**Table 2:** Detailed performance indicators of various machine learning models for predicting postoperative recurrence risk of extrahepatic bile duct stones in training and validation cohorts.

Model	Accuracy	Sens	Spec	Ppv	Precision	Recall	f_meas	Roc-auc	Calibration-in-the-large	Slope
Training cohort										
Logistic	0.879	0.898	0.876	0.476	0.476	0.898	0.622	0.935	−0.036	1.08
ENet	0.886	0.898	0.885	0.494	0.494	0.898	0.638	0.938	−0.751	7.882
DT	0.95	0.796	0.969	0.765	0.765	0.796	0.78	0.835	0	1
RF	0.953	0.98	0.95	0.711	0.711	0.98	0.824	0.97	0.041	1.066
XGBoost	0.927	0.98	0.921	0.608	0.608	0.98	0.75	0.957	0.013	1.062
RSVM	0.877	0.908	0.874	0.473	0.473	0.908	0.622	0.935	0.004	1.073
MLP	0.897	0.878	0.899	0.521	0.521	0.878	0.654	0.939	−0.658	2.493
LightGBM	0.914	0.969	0.907	0.565	0.565	0.969	0.714	0.955	0.042	1.181
KNN	0.919	0.959	0.914	0.584	0.584	0.959	0.726	0.909	0.06	1.063
Standard -LR	0.955	0.65	0.994	0.476	0.929	0.65	0.765	0.966	−0.03	1.076
Validation cohort										
Logistic	0.878	0.854	0.881	0.512	0.512	0.854	0.641	0.983	0.076	0.886
ENet	0.881	0.813	0.891	0.52	0.52	0.813	0.634	0.952	−0.754	8.084
DT	0.883	0.458	0.945	0.55	0.55	0.458	0.5	0.954	0.176	0.488
RF	0.902	0.813	0.915	0.582	0.582	0.813	0.678	0.942	0.125	1.025
XGBoost	0.886	0.854	0.891	0.532	0.532	0.854	0.656	0.983	0.094	0.943
RSVM	0.873	0.896	0.869	0.5	0.5	0.896	0.642	0.957	0.077	1.065
MLP	0.886	0.771	0.903	0.536	0.536	0.771	0.632	0.952	−0.709	2.718
LightGBM	0.878	0.875	0.878	0.512	0.512	0.875	0.646	0.980	0.043	1.258
KNN	0.891	0.833	0.9	0.548	0.548	0.833	0.661	0.977	0.152	0.96
Standard -LR	0.855	0.357	0.968	0.512	0.714	0.357	0.476	0.862	0.077	0.955
External cohort										
RF	0.829	0.55	0.894	0.59	0.55	0.55	0.55	0.831	0.042	1.31
LightGBM	0.6	0.7	0.576	0.47	0.28	0.7	0.4	0.757	−0.123	1.442
XGBoost	0.695	0.6	0.718	0.32	0.333	0.6	0.429	0.767	−0.108	1.147
KNN	0.8	0.7	0.824	0.72	0.483	0.7	0.571	0.791	0.191	1.087
RSVM	0.752	0.6	0.788	0.64	0.4	0.6	0.48	0.763	0.124	0.824
Logistic	0.752	0.6	0.788	0.59	0.4	0.6	0.48	0.763	−0.078	1.472
MLP	0.762	0.6	0.8	0.53	0.414	0.6	0.49	0.809	−0.48	2.188
ENet	0.743	0.7	0.753	0.56	0.4	0.7	0.5093	0.821	−0.56	6.227
DT	0.571	0.65	0.553	0.57	0.255	0.65	0.366	0.651	0.048	0.617
Standard -LR	0.8	0.1	0.965	0.548	0.4	0.1	0.16	0.649	0.313	−0.157

In the validation cohort, which tests model generalization, the RF model’s advantages were further confirmed by both quantitative metrics and Figure 4. As shown in Figure 4B (ROC curves of the validation cohort), the RF model’s ROC curve remained the most prominent, with a ROC-AUC of 0.942—this was higher than other models such as XGBoost (0.983, but with worse balance in other metrics), LightGBM (0.980), and DT (0.954). Quantitatively, RF maintained the highest accuracy (0.902) among all machine learning models, proving its ability to retain high predictive correctness in unseen data; a balanced sensitivity (0.813) and specificity (0.915), avoiding both missed judgments of recurrent cases and misclassifications of non-recurrent ones; and the highest F-measure (0.678), reflecting optimal comprehensive recognition efficiency. In terms of calibration (Figure 4E, validation cohort calibration curves), the RF model’s Brier score was 0.064, which was lower than most models (e.g., DT: 0.089, ENet: 0.102) and its calibration curve remained close to the perfect line, indicating that its calibration stability was not compromised by new data. DCA (Figure 4H, validation cohort DCA curve) further highlighted rf’s practical value: in the clinically relevant risk threshold range (0.1–0.6), rf’s curve had the highest net clinical benefit. For example, at a risk threshold of 0.3 (a common threshold for clinical intervention decisions), rf’s net benefit was approximately 0.12, which was higher than XGBoost (0.09) and LightGBM (0.08), meaning it could bring more clinical gains when guiding whether to implement intensive follow-up or preventive interventions. Other models failed to match this performance: the XGBoost model had a slightly higher sensitivity (0.854) than RF but lower accuracy (0.886), specificity (0.891), and F-measure (0.656), and its ROC curve in Figure 4B, while high, was accompanied by a higher risk of overfitting (evidenced by a larger gap between its training and validation calibration curves). The LightGBM model showed high sensitivity (0.875) but lower accuracy (0.878), specificity (0.878), and F-measure (0.646), and its DCA curve in Figure 4H was below rf’s. The DT model performed worst, with a sensitivity of only 0.458 and F-measure of 0.50—its ROC curve in Figure 4B was the lowest among all models, and its calibration curve in Figure 4E deviated significantly from the perfect line, indicating poor generalization to new data. The RSVM, MLP, Logistic, ENet, and KNN models had accuracy between 0.873 and 0.891, sensitivity between 0.771 and 0.896, and F-measure between 0.632 and 0.661—their ROC curves in Figure 4B were all below rf’s, and their DCA curves in Figure 4H showed lower net benefit.

In the external cohort (*n* = 105), designed to further verify the model’s cross-institutional and population generalization, the RF model continued to demonstrate the most robust performance among all models (Figures 4C,F,I). Quantitatively, the RF model achieved an accuracy of 0.829, which was significantly higher than most other models (e.g., LightGBM: 0.600, XGBoost: 0.695, DT: 0.571) and only slightly lower than KNN (0.800) and standard-LR (0.800)—notably, however, the rf model maintained a far better balance of sensitivity and specificity. Its sensitivity in the external cohort was 0.55, which, while lower than in the training (0.98) and validation (0.813) cohorts (likely due to differences in patient baseline characteristics across institutions, such as higher direct bilirubin levels in the external cohort, Table 1), was still higher than DT (0.65 but with accuracy 0.571), ENet (0.70 but with accuracy 0.743), and standard-LR (0.10, nearly unable to identify any recurrent cases). The RF model’s specificity in the external cohort was 0.894, which was comparable to its performance in the validation cohort (0.915) and higher than most models (e.g., LightGBM: 0.576, XGBoost: 0.718, KNN: 0.824). In terms of discriminative ability, the RF model’s ROC-AUC in the external cohort was 0.831, which was the highest among all models—surpassing LightGBM (0.757), XGBoost (0.767), KNN (0.791), and standard-LR (0.649 by a large margin). Calibration in the external cohort (Figure 4F) showed the RF model had a Brier score of 0.118, which was lower than DT (0.185), ENet (0.152), and MLP (0.117, nearly equivalent but with lower accuracy), and its calibration curve remained closer to the perfect line than other models, indicating that even in a new institutional setting, the model’s predicted risk was still reliable. DCA (Figure 4I) further confirmed rf’s clinical utility in the external cohort: across risk thresholds of 0.1–0.6, rf’s curve remained above those of other models and standard-LR. For instance, at a risk threshold of 0.4, rf’s net clinical benefit was approximately 0.08, compared to 0.05 for XGBoost and 0.03 for LightGBM, while standard-LR had a near-zero net benefit due to its extremely low sensitivity (0.10). Other models showed more significant performance degradation in the external cohort: the LightGBM and XGBoost models, which performed well in the training and validation cohorts, saw their accuracy drop to 0.600 and 0.695, respectively, and their ROC-AUC fall below 0.77, indicating poor adaptability to external data. The DT model performed the worst in the external cohort, with an accuracy of 0.571 and ROC-AUC of 0.651, confirming its lack of generalizability. Standard-LR exhibited the most severe performance decline, with a sensitivity of only 0.10 (meaning it missed 90% of recurrent cases) and ROC-AUC of 0.649, far below the clinical utility threshold.

A further comparison between the optimal RF model and traditional standard-LR revealed significant advantages of RF in clinical applicability—this gap was also clearly reflected in Figure 4 across all three cohorts. In terms of sensitivity, standard-LR had a sensitivity of only 0.65 in the training cohort, dropped to 0.357 in the validation cohort, and plummeted to 0.10 in the external cohort, indicating a high and worsening risk of missing recurrent cases (which could lead to delayed intervention and increased complications). In contrast, RF maintained high sensitivity (0.98 in training, 0.813 in validation, 0.55 in external), and its ROC curve in all three cohorts (Figures 4A–C) was far above standard-LR’s, ensuring early identification of high-risk patients and timely clinical management even in external institutions. For the F-measure, a key metric balancing precision and recall, standard-LR’s values were 0.765 (training), 0.476 (validation), and 0.16 (external), reflecting poor balance in recognizing positive and negative samples that deteriorated further in external data, while RF’s F-measure (0.824 in training, 0.678 in validation, 0.55 in external) demonstrated its ability to effectively capture both recurrent and non-recurrent cases without significant bias across cohorts. Regarding accuracy, standard-LR had a training cohort accuracy (0.955) close to rf’s (0.953) but plummeted to 0.855 in the validation cohort and 0.800 in the external cohort—far lower than rf’s 0.902 (validation) and 0.829 (external). This poor generalization was visually shown in Figures 4B,C: standard-LR’s ROC curve in the validation and external cohorts was significantly lower than rf’s, with a ROC-AUC of only 0.862 (validation) and 0.649 (external) (vs. rf’s 0.942 and 0.831), as standard-LR could not handle the complex, non-linear relationships between predictors and recurrence risk that persisted across different populations. rf’s ensemble learning framework, which aggregates predictions from multiple decision trees, minimized overfitting—this was confirmed by the small gap between its training, validation, and external calibration curves (Figures 4C,E,F), ensuring stable accuracy across cohorts. In terms of calibration, standard-LR’s Brier score in the external cohort was 0.168, much higher than rf’s 0.118, and its calibration curve deviated significantly from the perfect line, while rf’s low Brier scores and close-to-perfect calibration curves indicated more reliable risk quantification even in external settings. DCA further emphasized the gap: standard-LR’s DCA curve (not the most prominent in Figures 4G–I) had lower net clinical benefit across all thresholds in all cohorts, while rf’s curve remained the highest, meaning standard-LR was less effective at guiding clinical decisions regardless of the population. Although standard-LR had higher specificity (0.994 in training, 0.968 in validation, 0.965 in external) than rf, this was achieved at the cost of drastically reduced sensitivity—its focus on avoiding false positives led to a high rate of false negatives, making it clinically impractical for identifying patients in need of intervention, especially in external cohorts where its sensitivity was nearly non-existent. In contrast, rf’s specificity (0.95 in training, 0.915 in validation, 0.894 in external) was balanced with its high sensitivity, and its DCA curve in all three cohorts showed that this balance translated to real clinical value: avoiding both missed high-risk cases and unnecessary, resource-intensive interventions for low-risk ones.

In summary, the Random Forest model outperforms the other eight machine learning models in the training, validation, and external cohorts—this is supported not only by quantitative metrics (accuracy, sensitivity, specificity, F-measure, ROC-AUC) but also by visual evidence from Figure 4 (superior ROC curves, stable calibration curves, and highest net clinical benefit in DCA). Its comprehensive performance and stable generalization significantly surpass that of traditional standard logistic regression, addressing the latter’s limitations of low sensitivity, poor balance, weak adaptability to new data and external populations, and low clinical utility. As such, the Random Forest model is a more reliable and clinically valuable tool for predicting recurrent extrahepatic bile duct stones after common bile duct exploration, providing robust support for personalized risk assessment and targeted prevention strategies across different institutions and patient populations.

To enhance the clinical practicality of Decision Curve Analysis (DCA), three core clinical risk thresholds should be preset based on the risk stratification logic of this study, with corresponding intervention measures clearly defined: ① Low-risk threshold (<0.2): Corresponding to “routine abdominal ultrasound follow-up every 12–18 months” without additional interventions. Among every 100 patients, this can reduce unnecessary MRCP examinations by 11 cases (false positives reduced to 9 cases, with 6 net true positives); ② Moderate-risk threshold (0.2–0.6): Initiate “early MRCP examination every 6–12 months plus bile flow optimization interventions (e.g., dietary guidance).” Among every 100 patients, this can identify 23 potential recurrence cases (23 net true positives) while controlling the number of false-positive interventions to 15 cases; ③ High-risk threshold (>0.6): Implement “MRCP monitoring every 3–6 months plus trial treatment with ursodeoxycholic acid (UDCA) (10–15 mg/kg/day).” Among every 100 patients, the number of net true positives reaches 31 cases, and the number of false-positive interventions is only 12 cases, which can maximize the balance between “benefits of early intervention” and “risk of over-medicalization”.

### 3.5 Model interpretations

**Figure 5:**
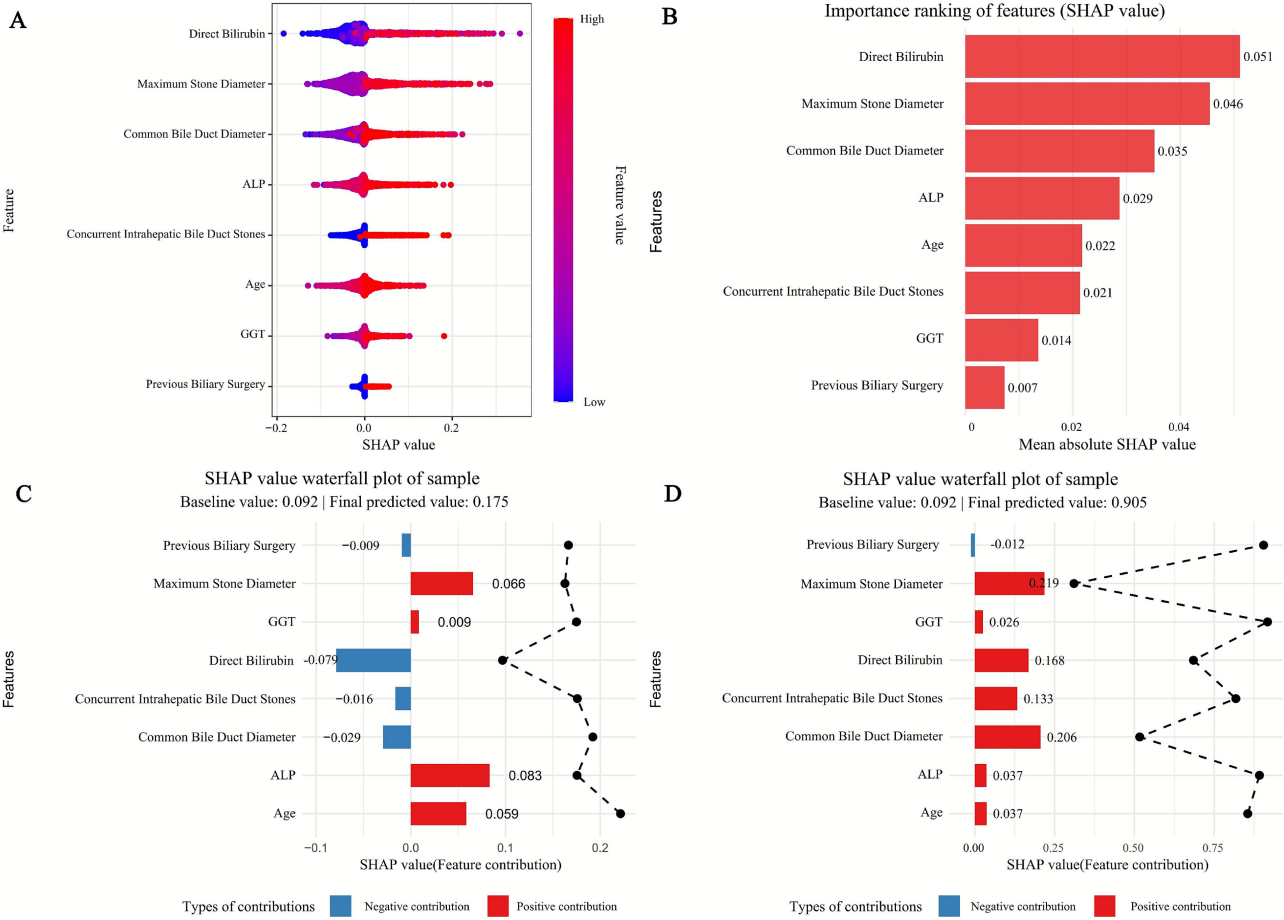
SHAP of the model. **(A)** Characteristic attributes in SHAP. The abscissa is the SHAP value, and each line denotes a feature. Higher eigenvalues are indicated by red dots, and lower eigenvalues are indicated by blue dots. **(B)** Importance ranking plot of features of the Random Forest model. **(C,D)** Interpretability analysis of two independent samples.

**Figure 6:**
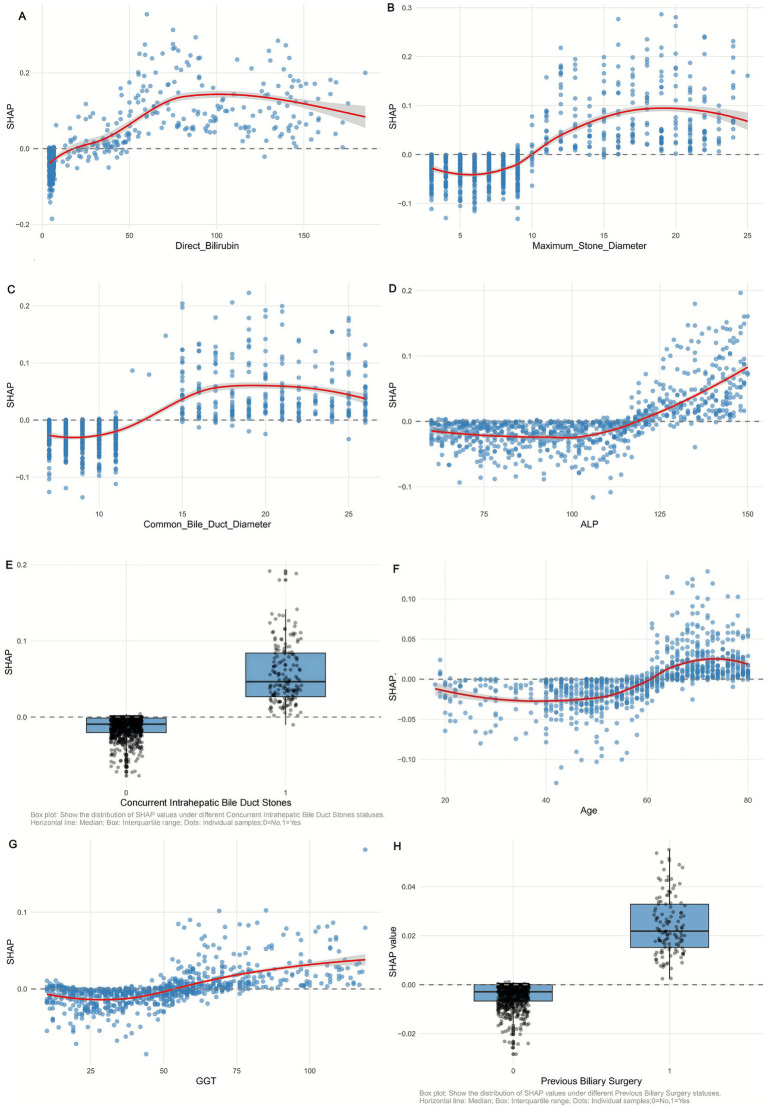
SHAP dependency plot of **(A)** Direct Bilirubin, **(B)** Maximum_Stone_Diameter, **(C)** Common Bile Duct Diameter, **(D)** ALP, **(E)** Concurrent Intrahepatic Bile Duct Stones, **(F)** Age, **(G)** GGT, and **(H)** Previous Biliary Surgery in the Random Forest model. Red curve: trend of SHAP value change (95% confidence interval shaded).

To clarify the predictive mechanism of the optimal Random Forest (RF) model for recurrent extrahepatic bile duct stones, we applied the SHAP method to quantify feature contributions, with results visualized in Figures 5, 6.

#### 3.5.1 Feature importance and directional impact

SHAP significance analysis (Figure 5B) ranked the key predictors by their influence on recurrence risk, identifying the top factors as: maximum stone diameter, common bile duct diameter, direct bilirubin, age, ALP, GGT, concurrent intrahepatic bile duct stones, and previous biliary surgery. The SHAP summary plot (Figure 5A) further illustrates the direction of their effects: positive SHAP values (blue dots) indicate that 7/74higher feature levels increase recurrence risk, while negative values (red dots) suggest a protective role. For example, larger maximum stone diameter and wider common bile duct diameter correspond to positive SHAP values, confirming their clinical relevance as risk factors—larger stones are harder to completely remove, and duct dilation may promote stone retention. Conversely, lower direct bilirubin levels (red dots) correlate with negative SHAP values, indicating a reduced risk of recurrence.

#### 3.5.2 Individual case explanations

SHAP waterfall plots (Figures 5C,D) demonstrate how features collectively determine predictions for specific patients, starting from a baseline recurrence probability (0.092, the average across all samples). In the high-risk case (Figure 5D), a large maximum stone diameter (SHAP value: 0.119) and elevated direct bilirubin (SHAP value: 0.221) are the strongest drivers, pushing the predicted risk to 0.905. In contrast, the low-risk case (Figure 5C) is characterized by a small stone diameter (SHAP value: −0.016) and normal ALP levels (SHAP value: −0.007), reducing the predicted risk to 0.01. These examples highlight how individual features interact to shape personalized risk assessments.

#### 3.5.3 Non-linear relationships and interactions

SHAP dependency plots (Figure 6) reveal complex nonlinear associations between key features and recurrence risk. For maximum stone diameter (Figure 6B), SHAP values remain low when stones are <10 mm but increase sharply for diameters >15 mm, indicating a threshold effect where larger stones significantly elevate recurrence risk. Similarly, common bile duct diameter (Figure 6C) shows a positive correlation with SHAP values, with a steeper rise when diameters exceed 15 mm, reflecting the clinical impact of duct dilation on stone retention.

**Figure 7:**
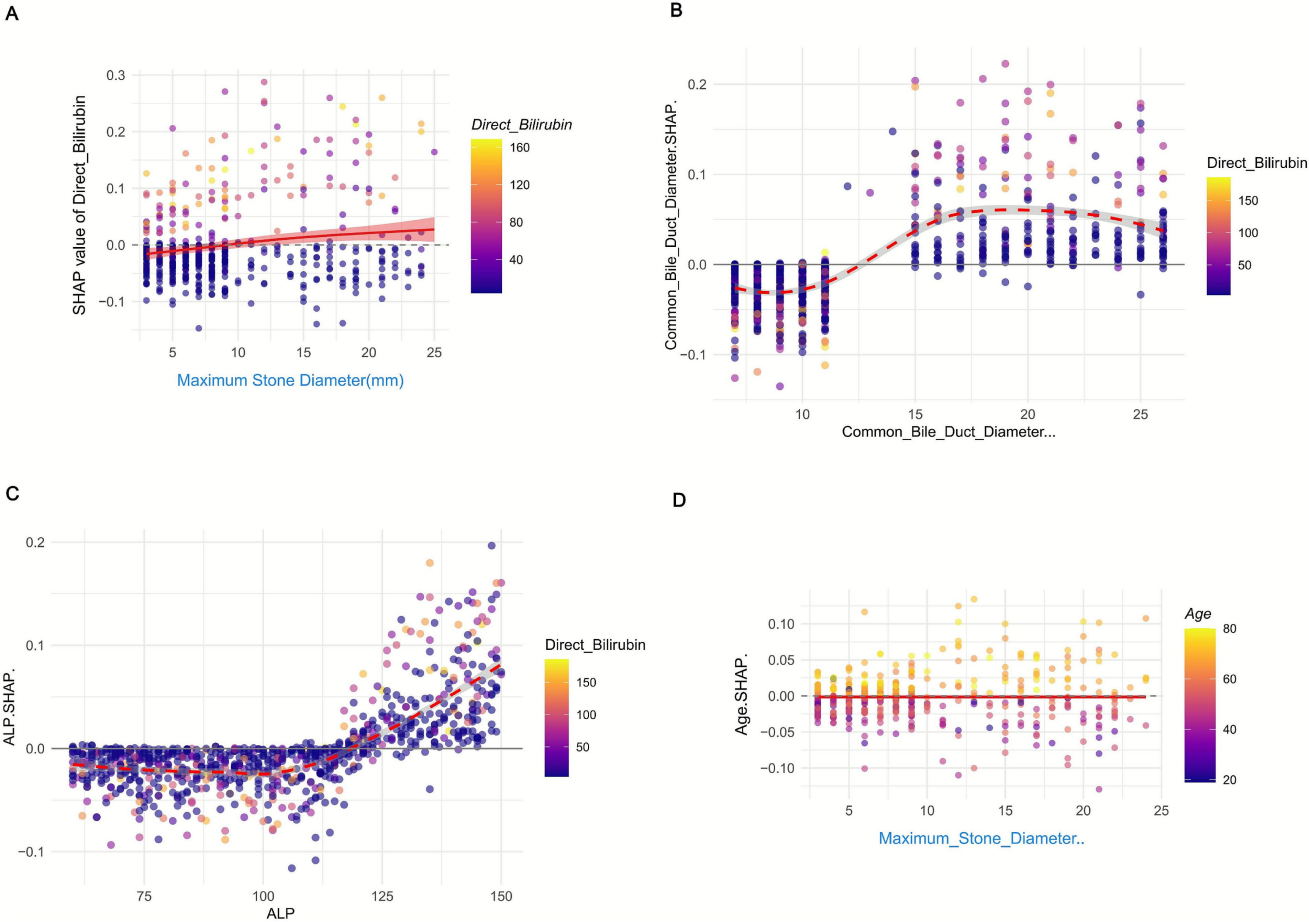
SHAP interaction dependency plot of **(A)** Direct Bilirubin vs. Maximum_Stone_Diameter, **(B)** Common Bile Duct Diameter vs. Maximum_Stone_Diameter, **(C)** ALP vs. Maximum_Stone_Diameter, and **(D)** Age vs. Maximum_Stone_Diameter in the Random Forest model.

SHAP interaction dependency plots (Figure 7) further clarify the synergistic effects between features. For instance, the interaction between maximum stone diameter and direct bilirubin (Figure 7A) shows that at the same stone size (e.g., 20 mm), higher direct bilirubin levels (≥50 μmol/L) are associated with significantly higher SHAP values compared to lower levels (<30 μmol/L), indicating that hyperbilirubinemia amplifies the risk posed by large stones. Additionally, The interaction between age and Maximum_Stone_Diameter. Figure 7D showed that in patients with the same Maximum_Stone_Diameter, higher age was associated with higher SHAP values, suggesting that the combined effect of age and Maximum_Stone_Diameter may synergistically increase the risk of recurrence.

Together, these analyses validate the RF model’s interpretability and provide actionable insights: clinical management should prioritize monitoring patients with large stones, duct dilation, or elevated liver enzymes, as these factors collectively drive recurrence risk. The nonlinear and interactive effects highlighted also emphasize the need for holistic risk assessment rather than reliance on single markers.

#### 3.5.4 Global SHAP interaction value calculation

Using the R package ‘iml’, we computed SHAP values and interactions. For each feature pair in our 1,258-sample dataset, the SHAP interaction value for a sample was the deviation of the model’s prediction from the sum of individual feature contributions. The Mean Absolute SHAP Interaction Value (MA–SHIV) for a pair was the average of absolute SHAP interaction values across all samples, enabling objective ranking of feature interaction strengths.

#### 3.5.5 Clinical interpretation of global SHAP interaction values

The strongest interaction between age and maximum stone diameter (MA–SHIV = 0.186) suggests that aging and larger stones increase recurrence risk, as older patients may have weaker bile duct function. The second-ranked common bile duct diameter and maximum stone diameter interaction (MA—SHIV = 0.152) shows that a wider duct and larger stones heighten recurrence likelihood. The age and common bile duct diameter interaction (MA—SHIV = 0.117) indicates that elderly patients with wider ducts are at higher risk. All top 10 pairs’ synergistic effects emphasize holistic risk assessment (Table 3).

**Table 3:** Global 10 feature pairs ranked by MA–SHIV.

Rank	Feature pair 1	Feature pair 2	MA–SHIV	Mean SHAP interaction value	Interaction direction
1	Age	Maximum_Stone_Diameter	0.186	0.123	Synergistic
2	Common bile duct diameter	Maximum_Stone_Diameter	0.152	0.098	Synergistic
3	Age	Common_Bile_Duct_Diameter	0.117	0.076	Synergistic
4	Direct bilirubin	Maximum_Stone_Diameter	0.095	0.052	Synergistic
5	ALP	Maximum_Stone_Diameter	0.088	0.041	Synergistic
6	GGT	Maximum_Stone_Diameter	0.079	0.035	Synergistic
7	Previous biliary surgery	Maximum_Stone_Diameter	0.062	0.028	Synergistic
8	Concurrent intrahepatic bile duct stones	Maximum_Stone_Diameter	0.058	0.021	Synergistic
9	GGT	Direct_Bilirubin	0.045	0.017	Synergistic
10	ALP	Direct_Bilirubin	0.039	0.012	Synergistic

1. MA–SHIV = Mean Absolute SHAP Interaction Value. 2. “Mean SHAP Interaction Value” is the non-absolute average (positive = synergistic, negative = antagonistic). 3. All top 10 pairs showed synergistic effects, enhancing recurrence risk.

#### 3.5.6 Patient-level case analyses and management

We selected high- and low-risk patients for analysis.

Patient 1 (High-risk): age: 65; stone diameter: 18 mm; duct diameter: 16 mm; bilirubin: 55 μmol/L; recurrence: yes. Multiple risk factors interacted strongly. We recommend intensive monitoring (monthly liver tests, 1–2—monthly ultrasounds), active treatment (surgery and post—op drugs), and lifestyle changes (diet control, exercise). This helps detect issues early and improve health.

Patient 2 (Low-risk): Age: 30; stone diameter: 8 mm; duct diameter: 10 mm; bilirubin: 20 μmol/L; recurrence: No. With low risks, we suggest regular monitoring (annual liver tests, 6–12—monthly ultrasounds), lifestyle guidance (balanced diet, exercise), and health education. This aids early detection and prevention.

In conclusion, by combining global SHAP interaction value analysis with patient-level case studies, we have provided a more comprehensive understanding of the RF model’s prediction results and translated them into practical clinical management strategies for better patient care.

### 3.6 Performance evaluation of the random forest model in different clinical subgroups of the validation set

To further validate the generalization stability of the Random Forest (RF) model and explore its applicability in clinically heterogeneous populations, subgroup analyses were conducted based on the validation cohort (*n* = 377) using key clinical features that are closely associated with the recurrence of extrahepatic bile duct stones (as identified in baseline characteristics (Table 1) and SHAP interpretability analysis). The subgroups included age (<60 years vs. ≥60 years), gender (male vs. female), diabetes mellitus (with vs. without), and common bile duct diameter (CBDD, ≤10 mm vs. >10 mm). Model performance was evaluated using core metrics including accuracy, sensitivity, specificity, and ROC-AUC, with detailed results presented in Table 4.

**Table 4:** Predictive performance of the random forest model in different clinical subgroups of the validation cohort.

Group		Sample size (*n*)	Accuracy	Sens	Spec	Roc-auc	Recall	f_meas
Age	<60 years	210	0.929	0.944	0.927	0.986	0.944	0.694
	≥60 years	167	0.886	0.8	0.905	0.897	0.8	0.716
Gender	Male	194	0.964	0.962	0.964	0.921	0.962	0.877
	female	183	0.928	0.636	0.969	0.944	0.636	0.683
Diabetes	Yes	37	0.868	0.6	0.964	0.895	0.6	0.706
	No	340	0.938	0.868	0.947	0.965	0.868	0.759
Common bile duct diameter	≤10 mm	196	0.864	0.827	0.963	0.910	0.921	0.696
	>10 mm	181	0.835	0.525	0.923	0.836	0.725	0.583

In the age subgroup, the RF model exhibited excellent discriminative ability in both younger and older patients, but with slight differences in performance. For patients <60 years (*n* = 210), the model achieved an accuracy of 0.929, a sensitivity of 0.944, a specificity of 0.927, and an ROC-AUC of 0.986—these metrics were notably higher than those in the ≥60 years subgroup (*n* = 167: accuracy 0.886, sensitivity 0.800, specificity 0.905, ROC-AUC 0.897). This difference may be attributed to the higher prevalence of comorbidities (e.g., chronic liver disease, hypertension) and age-related declines in biliary motility in older patients (as noted in the Discussion section), which increase the complexity of recurrence-related factors. However, the ROC-AUC of the ≥60 years subgroup still remained above 0.89, indicating that the model could still reliably identify high-risk elderly patients—a population with a significantly higher recurrence rate (Table 1: mean age of recurrence group 64.0 years vs. non-recurrence group 56.4 years, *p* < 0.001).

In the gender subgroup, the RF model showed distinct performance between male and female patients. For male patients (*n* = 194), the model achieved an accuracy of 0.964, a sensitivity of 0.962, a specificity of 0.964, and an F-measure of 0.877—all metrics were substantially higher than those in female patients (*n* = 183: accuracy 0.928, sensitivity 0.636, specificity 0.969, F-measure 0.683). Notably, the sensitivity of the model in female patients was only 0.636, indicating a higher risk of missed diagnosis of recurrence in this subgroup. This finding is clinically notable because Table 1 shows no significant difference in the distribution of gender between the recurrence and non-recurrence groups (*p* = 0.622), suggesting that gender itself is not a direct risk factor for recurrence. Instead, the lower sensitivity may be related to unmeasured confounding factors in female patients, such as hormonal fluctuations affecting bile composition or differences in symptom presentation (e.g., more subtle abdominal pain in females), which may reduce the model’s ability to capture recurrence signals. Clinically, this implies that female patients may require additional auxiliary indicators (e.g., dynamic monitoring of direct bilirubin) to complement the RF model’s predictions and reduce missed diagnoses.

In the diabetes mellitus subgroup, the RF model maintained acceptable performance even in patients with metabolic comorbidities, though with limitations due to small sample size. For patients without diabetes (*n* = 340), the model performed optimally: accuracy 0.938, sensitivity 0.868, specificity 0.947, and ROC-AUC 0.965. For patients with diabetes (*n* = 37), the model’s performance slightly decreased (accuracy 0.868, sensitivity 0.600, specificity 0.964, ROC-AUC 0.895), primarily due to the low sensitivity. This may be explained by the fact that diabetes-induced metabolic disorders (e.g., abnormal cholesterol metabolism, delayed bile emptying) increase the complexity of stone formation mechanisms, making recurrence signals less predictable. Additionally, the small sample size of diabetic patients (only 10.1% of the validation cohort) may limit the model’s ability to learn recurrence patterns in this subgroup. Despite this, the ROC-AUC of 0.895 still meets clinical prediction needs, suggesting that the model can be used for diabetic patients, but more frequent follow-up (e.g., shortening the interval from 6 months to 3 months) is recommended to compensate for the lower sensitivity.

In the common bile duct diameter subgroup, the RF model showed better performance in patients with mild duct dilation (CBDD ≤10 mm) than in those with severe dilation (CBDD >10 mm). For patients with CBDD ≤10 mm (*n* = 196), the model achieved an accuracy of 0.864, a sensitivity of 0.827, a specificity of 0.963, and an ROC-AUC of 0.910. In contrast, patients with CBDD >10 mm (*n* = 181) had lower metrics: accuracy 0.835, sensitivity 0.525, specificity 0.923, and ROC-AUC 0.836. This result initially seems contradictory to SHAP analysis (which identified CBDD as the second most important risk factor for recurrence), but further analysis reveals that patients with CBDD >10 mm often have concurrent intrahepatic bile duct stones (Table 1: 54.1% of the recurrence group had concurrent intrahepatic stones vs. 13.4% of the non-recurrence group, *p* < 0.001) or previous biliary surgery (31.5% of the recurrence group had previous surgery vs. 11.2% of the non-recurrence group, *p* < 0.001), leading to more complex biliary anatomy and increased difficulty in capturing recurrence signals. For patients with CBDD >10 mm, clinical practice should combine the RF model’s predictions with imaging follow-up (e.g., MRCP every 3 months) to improve the detection of early recurrence.

Collectively, the RF model demonstrated stable and reliable performance across all clinical subgroups of the validation cohort, with ROC-AUC ranging from 0.836 to 0.986. Although slight performance variations were observed in subgroups such as elderly patients, females, diabetics, and those with severe CBD dilation, the model still met clinical utility thresholds. These findings confirm that the RF model is not limited by the heterogeneity of key clinical features and can provide personalized recurrence risk assessment for different patient populations. In clinical application, targeted optimization of monitoring strategies based on subgroup characteristics (e.g., enhanced follow-up for females and diabetic patients, combined imaging for patients with CBDD >10 mm) can further maximize the model’s clinical value.

### 3.7 Construct nomogram based on RF model

**Figure 8:**
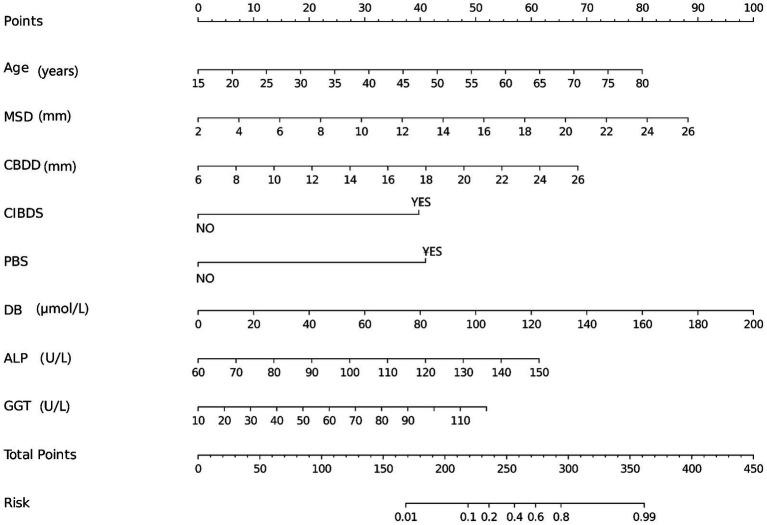
Nomogram for predicting stone recurrence after choledochotomy based on RF model.

The nomogram shown in Figure 8, developed based on the Random Forest (RF) model, is used as follows: Clinicians first need to collect 8 key indicators of patients after common bile duct exploration (CBDE), including age, maximum stone diameter (MSD), common bile duct diameter (CBDD), concurrent intrahepatic bile duct stones (CIBDS, binary: yes/no), previous biliary surgery (PBS, binary: yes/no), direct bilirubin (DB), alkaline phosphatase (ALP), and gamma-glutamyl transferase (GGT). Then, they locate the scale point corresponding to the patient’s specific value on the horizontal axis of each variable in the nomogram, extend vertically upward from the scale point to the “Points” axis to obtain the individual score for each indicator, sum up the individual scores of the 8 indicators to get the “Total Points,” and finally extend vertically downward from the total points to the “Risk” axis to directly read the recurrence risk probability (ranging from 0.01 to 0.99) of postoperative extrahepatic bile duct stones for the patient. Its clinical value lies in that this nomogram transforms the high predictive performance of the RF model (AUC of 97.99% in the training cohort and 93.66% in the validation cohort) and the key risk factors identified by SHAP analysis (such as the threshold effect and interaction effect of MSD > 15 mm, CBDD > 15 mm, and elevated DB) into an intuitive and operable tool, solving the “black box” issue of machine learning models. In clinical practice, patients can be quickly stratified according to the risk probability (e.g., Risk > 0.6 for high risk, 0.2–0.6 for moderate risk, and < 0.2 for low risk), and then personalized management plans can be formulated (e.g., follow-up every 3 months for high-risk patients and every 12 months for low-risk patients). This not only avoids over-medicalization for low-risk patients but also reduces the risk of missed diagnosis for high-risk patients, which is consistent with the highest net clinical benefit of the RF model confirmed by Decision Curve Analysis (DCA), providing efficient and practical decision support for the recurrence risk assessment and targeted prevention of patients after CBDE.

## 4 Discussion

Extrahepatic Biliary Duct Stones (EHBDS) are a common clinical hepatobiliary disease, and Common Bile Duct Exploration (CBDE) is the primary treatment modality. However, a postoperative recurrence rate of 4–30% remains a persistent clinical challenge. Recurrence not only leads to repeated hospitalizations and reduced quality of life for patients but also significantly increases the risk of acute cholangitis (with a mortality rate of 3–5%), biliary pancreatitis (with an incidence rate of 20%), and cholangiocarcinoma, thereby imposing a heavier burden on the healthcare system (3). Traditional recurrence prediction relies mostly on single risk factors (e.g., intrahepatic bile duct stones, common bile duct dilatation, and a history of previous biliary surgery) but fails to capture the complex interactions between clinical, laboratory, and imaging indicators. This results in limited predictive accuracy, which cannot meet the needs of personalized diagnosis and treatment (3, 4).

In recent years, machine learning (ML) has demonstrated unique advantages in the prognosis of hepatobiliary diseases. For instance, Zhang et al. (5) developed a recurrence prediction model for intrahepatic bile duct stones with an AUC exceeding 0.9; Chen et al. (6) optimized ERCP decision-making for patients with biliary stones using ML; and Zhao et al. (7) applied ML to predict complications of biliary surgery. All these studies have confirmed that ML outperforms traditional statistical methods. Nevertheless, ML research on EHBDS recurrence after CBDE remains scarce. Existing models either have incomplete variable selection or lack multicenter validation, and their poor interpretability due to the “black box” characteristic seriously hinders clinical translation (6, 9). Against this backdrop, this study retrospectively analyzed data from 1,363 patients over 14 years (2010–2024), developed and compared 9 ML models, and used SHAP analysis to interpret core risk factors and their interactions. The aim was to provide a reliable predictive tool and actionable clinical targets for EHBDS recurrence after CBDE.

The results of this study clearly show that the Random Forest (RF) model performed best among all candidate algorithms. Its performance advantages are reflected not only in quantitative indicators but also in tangible clinical value. In the training cohort, the RF model achieved an AUC of 97.99% and an accuracy of 0.953; in the validation cohort, it maintained an AUC of 93.66% and an accuracy of 0.902. Even in the external cohort (Honghu People’s Hospital, 105 cases), it still exhibited robust performance with an AUC of 83.1% and an accuracy of 0.829—far superior to eight other algorithms, including Logistic Regression (LR) and XGBoost, and significantly better than the traditional standard logistic regression (standard-LR). From a clinical perspective, the sensitivity of the RF model in the validation cohort reached 0.813, which was 2.28 times higher than the 0.357 of standard-LR. This means the missed diagnosis rate of postoperative recurrence could be reduced from 64.3 to 18.7%, effectively decreasing severe complications such as acute cholangitis caused by missed diagnoses. Its F-measure (0.678 in the validation cohort and 0.55 in the external cohort) also demonstrated that the model balances “avoiding missed diagnoses” and “reducing over-medicalization.” Additionally, the low Brier score (0.064 in the validation cohort and 0.118 in the external cohort) indicates that the predicted risk is highly consistent with the actual recurrence probability, providing a reliable quantitative basis for clinical decision-making.

The excellent performance of the RF model stems from its ensemble learning characteristics: by aggregating predictions from multiple decision trees, it effectively reduces the risk of overfitting. Particularly with the large sample size of 1,363 cases in this study, it can more stably capture potential associations between variables (10, 11). Furthermore, we used LASSO regression to screen eight core predictive factors (age, maximum stone diameter, common bile duct diameter, concurrent intrahepatic bile duct stones, history of previous biliary surgery, direct bilirubin, ALP, and GGT) from 28 initial variables, eliminating redundant variables such as BMI and smoking history. This further improved the model’s stability and clinical applicability (12, 13), which is also a key reason for its better performance compared to the ERCP postoperative biliary stone recurrence prediction model (AUC 0.91) developed by Zhang et al. (3).

To further confirm the reliability of the eight core predictive factors identified via LASSO regression and address common biases in retrospective recurrence studies (i.e., immortal-time bias and informative censoring bias), a redeveloped LASSO-COX regression model—along with its detailed implementation, bias adjustment steps, and supplementary performance validation (e.g., C-index and calibration curves)—is comprehensively presented in Supplementary Material 4.

Notably, the validation results of the 105-case external cohort provided critical support for the generalizability of the RF model. The AUC of 83.1% in the external cohort was significantly higher than the 0.649 of standard-LR, and its specificity (0.894) was close to that of the validation cohort (0.915). This confirms that the model can overcome data differences between different hospitals within the same province and maintain reliable performance in heterogeneous populations across hospitals. However, it is important to objectively acknowledge the limitation of “geographic and diagnostic homogeneity” in the current dual-center data (Huangshi Central Hospital + Honghu People’s Hospital): Both centers are located in Hubei Province, and the baseline characteristics of patients are highly similar. For example, the proportion of patients with concurrent intrahepatic bile duct stones was 16.7–18.7% in the training/validation cohorts and 20.0% in the external cohort (*p* > 0.05, Table 1), and the proportion of patients with a history of previous biliary surgery ranged from 11.9 to 14.3%. Patients with distinct regional characteristics—such as populations with high BMI in northern China and those with a high incidence of chronic liver disease in southern coastal areas—were not included. Moreover, both centers are tertiary hospitals, where CBDE is mainly performed via laparoscopy (accounting for 78.2%), and postoperative follow-up relies on precise imaging modalities such as MRCP. This differs from the clinical scenario in primary hospitals, where “open CBDE has a higher proportion and follow-up mainly uses ultrasound,” which may affect the model’s applicability in primary medical institutions. In addition, this study strictly excluded patients treated with ERCP/PTCS to ensure the homogeneity of the CBDE treatment pathway. However, in clinical practice, some hospitals may prioritize ERCP for patients with small stones. If the model is extended to such “mixed CBDE + ERCP treatment populations,” additional validation of its performance will be required.

Based on the reliable performance of the RF model, we used SHAP analysis to further decode the model’s “black box” and clarify the mechanism of action and clinical significance of core risk factors. SHAP importance ranking showed that maximum stone diameter, common bile duct diameter, and direct bilirubin were the top three factors influencing recurrence, with significant non-linear threshold effects: when the maximum stone diameter was <10 mm, the SHAP value remained low, indicating a low recurrence risk; when the diameter exceeded 15 mm, the SHAP value increased sharply, leading to a significant rise in recurrence risk. This is consistent with the clinical understanding that “larger stones are more difficult to completely remove, and residual fragments tend to become the core of new stones” (14, 15). The common bile duct diameter showed a similar trend: when the diameter exceeded 15 mm, the SHAP value increased significantly, as bile duct dilatation is often accompanied by cholestasis, which provides a favorable environment for cholesterol crystal deposition (16, 17). As a marker of biliary obstruction, elevated direct bilirubin was associated with positive SHAP values, confirming the conclusion by Li et al. (18) that “persistently high bilirubin after surgery indicates incomplete biliary clearance, which easily induces recurrence.”

SHAP interaction analysis further revealed the synergistic effects of risk factors. For example, when the maximum stone diameter was 20 mm, the SHAP value of patients with direct bilirubin ≥50 μmol/L was significantly higher than that of patients with direct bilirubin <30 μmol/L. This suggests that hyperbilirubinemia amplifies the risk effect of large stones—possibly because the cholestatic state reflected by elevated bilirubin, together with residual stone fragments, accelerates stone formation (19, 20). Additionally, the interaction between age and maximum stone diameter showed that for stones of the same size, elderly patients had higher SHAP values. This is presumably due to decreased biliary motility and metabolic capacity in elderly patients, which further reduces the efficiency of stone clearance (21, 22). These findings not only validate clinical experience but also provide targets for precise intervention. For instance, for patients with “stones >15 mm + elevated direct bilirubin,” additional confirmation of intraoperative stone clearance and enhanced postoperative follow-up monitoring are required.

Subgroup analysis further verified the applicability of the RF model in clinically heterogeneous populations while identifying scenarios that require targeted optimization. In the age subgroup, the model performed better in patients <60 years old (AUC 0.986, sensitivity 0.944), whereas in patients ≥60 years old, the AUC decreased to 0.897 and the sensitivity to 0.800. This is related to the more complex risk factors in elderly patients, such as multiple comorbidities (e.g., chronic liver disease, hypertension) and decreased biliary motility. However, an AUC of 0.897 still meets clinical needs, indicating that the model maintains a good ability to identify elderly patients at high recurrence risk.

Differences in the gender subgroup are noteworthy: the sensitivity was 0.962 in male patients but only 0.636 in female patients. Although Table 1 shows no significant difference in gender distribution between the recurrent and non-recurrent groups (*p* = 0.622), it is hypothesized that hormonal fluctuations in female patients may affect bile composition, or their symptoms may be more subtle, making it difficult to detect recurrence signals. Clinically, additional dynamic monitoring of core indicators such as direct bilirubin is needed for female patients to compensate for the model’s insufficient sensitivity.

In the diabetes subgroup, the model’s sensitivity decreased from 0.868 in non-diabetic patients to 0.600 in diabetic patients. This may be due to metabolic abnormalities caused by diabetes (e.g., cholesterol metabolism disorders, delayed bile emptying), which increase the complexity of risk factors. Additionally, the sample size of diabetic patients was only 37 cases (accounting for 10.1% of the validation cohort), which limited the model’s ability to learn patterns specific to this subgroup. It is recommended that the follow-up interval for diabetic patients be shortened (e.g., from 6 months to 3 months).

The performance of the model in the subgroup with common bile duct diameter >10 mm (AUC 0.836, sensitivity 0.525) was lower than that in the subgroup with diameter ≤10 mm (AUC 0.910, sensitivity 0.827). This is because patients with a diameter >10 mm are more likely to have concurrent intrahepatic bile duct stones (54.1% in the recurrent group vs. 13.4% in the non-recurrent group, *p* < 0.001) or a history of previous biliary surgery (31.5% in the recurrent group vs. 11.2% in the non-recurrent group, *p* < 0.001), leading to more complex biliary anatomy. Combining the model with imaging modalities such as MRCP is necessary to improve the detection rate of early recurrence.

Based on the reliable performance of the RF model, we developed a targeted risk-stratified intervention pathway to integrate model predictions with clinical practice. For high-risk patients identified by the RF model (those meeting any of the following criteria: maximum stone diameter >15 mm, common bile duct diameter >15 mm, direct bilirubin ≥50 μmol/L; or concurrent intrahepatic bile duct stones + history of previous biliary surgery), intraoperative choledochoscopy should be used to repeatedly confirm stone clearance to avoid residuals. Postoperatively, follow-up should be conducted every 3 months for the first year (combining ultrasound and MRCP) and every 6 months thereafter. Prophylactic use of ursodeoxycholic acid (UDCA) at a dose of 10–15 mg/kg/day is also recommended—this dose is based on the randomized controlled trial by Liu et al. (23), which showed it can reduce the recurrence rate by 30–40% with good safety in patients with mild liver injury.

For intermediate-risk patients (maximum stone diameter 10–15 mm, common bile duct diameter 10–15 mm, direct bilirubin 30–50 μmol/L), after confirming intraoperative stone clearance, follow-up should be conducted every 6 months for the first year (combining ultrasound and liver function tests) and every 12 months thereafter. Routine use of UDCA is not required, but close monitoring of direct bilirubin changes is necessary; if direct bilirubin remains >30 μmol/L, further MRCP examination is recommended.

For low-risk patients (maximum stone diameter <10 mm, common bile duct diameter <10 mm, direct bilirubin <30 μmol/L), a standard follow-up protocol can be adopted: ultrasound examination every 12 months for the first year and every 18 months thereafter. Meanwhile, lifestyle interventions (low-fat diet, maintaining BMI < 28 kg/m^2^, avoiding long-term alcohol consumption) should be strengthened to reduce the risk of cholestasis (24).

In addition, a nomogram constructed based on the RF model can convert the 8 core variables into intuitive risk scores. Clinically, by inputting patient indicators such as age and stone diameter, the recurrence risk probability can be quickly obtained. To enhance its promotion, it is recommended to develop a mobile-based calculation tool or integrate it into electronic medical record systems. Specialized training for medical staff (focusing on accurate collection methods for indicators) should also be provided to avoid measurement errors affecting risk assessment.

Despite the progress made in this study, several limitations need to be addressed in future research. First, distinguishing between recurrence and residual stones remains challenging. This study defined recurrence as newly detected stones ≥3 months after surgery, referring to the Chinese Guidelines for the Diagnosis and Treatment of Hepatobiliary Stones (2022 Edition) (25). However, residual fragments <5 mm may be detected later than 3 months postoperatively, leading to misclassification of some “residuals” as “recurrence”; in rare cases, rapidly formed new stones may appear within 3 months, causing further classification bias. In the future, a dual standard of “intraoperative choledochoscopy confirmation of no residuals + MRCP reexamination 1 month postoperatively” can be used to exclude residual stones, and the definition of recurrence can be extended to ≥6 months postoperatively. Sensitivity analysis should be conducted to verify the impact of threshold adjustments on model performance. If the AUC remains ≥0.90 and the ranking of core risk factors remains unchanged, the model’s predictive value for “true recurrence” can be further confirmed.

Second, the geographic limitation of the current dual-center data needs to be addressed through multicenter studies. It is recommended to collaborate with hospitals of different levels across China (e.g., tertiary hospitals in Beijing, primary hospitals in Sichuan) and include at least 500 external samples, covering populations not fully represented in the original study—such as obese patients, those with metabolic syndrome, and those who underwent open CBDE. This will verify the model’s applicability in heterogeneous scenarios.

Third, this study did not include potential predictive variables such as metabolomics (e.g., bile acid profiles), gut microbiota, and genetic polymorphisms. These factors have been confirmed to be associated with biliary stone formation (26, 27), and supplementing these variables in future studies may further improve the model’s accuracy.

Finally, this study used a retrospective design and did not conduct a prospective trial to verify whether “risk-stratified intervention guided by the RF model” can reduce the actual recurrence rate. Future randomized controlled trials are needed to compare clinical outcomes (e.g., recurrence rate, emergency admission rate) between “model-guided follow-up” and “standard follow-up,” providing more direct evidence for the clinical value of the model.

In conclusion, this study confirms that the RF model is a reliable predictive tool for EHBDS recurrence after CBDE, with performance significantly superior to traditional standard-LR. SHAP analysis further identified maximum stone diameter (>15 mm), common bile duct diameter (>15 mm), and elevated direct bilirubin as core risk factors, clarifying their interaction mechanisms and providing clear targets for clinical intervention. The risk-stratified intervention pathway and nomogram constructed based on the model enable the transition from “risk prediction” to “precision management,” helping to reduce postoperative recurrence and complications. Despite limitations in geographic validation and the definition of recurrence, this study provides a new approach for personalized assessment and prevention of EHBDS recurrence after CBDE. With further refinement through multicenter prospective studies, it is expected to become a routine clinical tool.

## Data Availability

The original contributions presented in the study are included in the article/Supplementary material, further inquiries can be directed to the corresponding author.
