# Supplementary material for: Machine learning-based prediction of recurrent extrahepatic bile duct stones after common bile duct exploration: a comparative study of models and SHAP-driven interpretability analysis
Source: Front Med (Lausanne). 2025 Dec 12;12:1691519. doi: 10.3389/fmed.2025.1691519 (PMC12741112; doi:10.3389/fmed.2025.1691519)
Supplement: Supplementary file 1 [file Supplementary_file_1.docx]

Supplementary Material

**Follow-up Protocol for Recurrent Extrahepatic Bile Duct Stones After Common Bile Duct Exploration**

**1. Purpose of Follow-up**

This follow-up protocol aims to dynamically monitor the disease progression of patients with extrahepatic bile duct stones (EHBDS) after common bile duct exploration (CBDE), promptly detect signs of stone recurrence, implement stratified management based on key preoperative/intraoperative risk factors (e.g., maximum stone diameter, common bile duct diameter, direct bilirubin level) identified in the study, reduce the incidence of recurrence-related complications (such as acute cholangitis and biliary pancreatitis), and provide real-world data support for the optimization of postoperative intervention strategies and the iteration of prediction models.

**2. Target Population**

Patients with EHBDS who underwent CBDE at Huangshi Central Hospital (including its Puai Campus) between October 2010 and October 2024, meeting the following criteria:

1. Postoperatively, stone clearance was confirmed by clinical (symptoms, signs) and imaging examinations (abdominal ultrasound, magnetic resonance cholangiopancreatography [MRCP], etc.), and there were no uncontrolled diseases such as active biliary infection or severe liver dysfunction (e.g., ALT/AST > 3 times the upper limit of normal) at discharge.

2. Patients voluntarily cooperate with follow-up and have valid contact information (phone number, WeChat, or home address).

3. Patients with end-stage diseases that may affect the completeness of follow-up (e.g., advanced cholangiocarcinoma, severe liver failure) and those who were lost to follow-up within 3 months after surgery or died of non-stone-related diseases are excluded.

**3. Start and End Time of Follow-up**

1. Start Time: The first day after the patient’s postoperative discharge is set as the starting point of follow-up. On the day of discharge, the primary nurse will enter the patient’s information into the follow-up system and inform the patient of the follow-up plan simultaneously.

2. End Time: The follow-up ends at the time of any of the following events:

- Confirmation of EHBDS recurrence by imaging examinations (MRCP/endoscopic retrograde cholangiopancreatography [ERCP]/computed tomography [CT]), which must meet the recurrence definition of "at least 3 months after surgery".

- Death of the patient due to non-stone-related diseases (a death certificate or family confirmation record is required).

- For patients who have not experienced recurrence by the study cut-off date (October 2024), the cut-off date is regarded as the end time.

- Patients who explicitly refuse to continue follow-up, with the refusal time and reason recorded.

**4. Follow-up Frequency and Methods**

**4.1 Stratified Follow-up Frequency**

Based on the key risk factors screened by the Random Forest (RF) model in the study, patients are divided into three risk levels (high, medium, and low), and a differentiated follow-up frequency is implemented. The specific stratification criteria and frequency are as follows:

| **RiskLevel** | **StratificationCriteria**  **(MeetAnyOneCondition)** | **Follow-upFrequency**  **(Within1YearAfterSurgery)** | **Follow-upFrequency**  **(MoreThan1YearAfterSurgery)** |
| --- | --- | --- | --- |
| High Risk | 1. Maximum stone diameter > 15 mm; 2. Common bile duct diameter > 15 mm; 3. Direct bilirubin ≥ 50 μmol/L;4. Concurrent intrahepatic bile duct stones + previous biliary surgery history | Once every 3 months | Once every 6 months |
| Medium Risk | 1. Maximum stone diameter 10–15 mm; 2. Common bile duct diameter 10–15 mm; 3. Direct bilirubin 34–50 μmol/L;   4. Only concurrent intrahepatic bile duct stones or only previous biliary surgery history | Once every 6 months | Once every 12 months |
| Low Risk | 1. Maximum stone diameter < 10 mm; <br>2. Common bile duct diameter < 10 mm; <br>3. Direct bilirubin < 34 μmol/L; <br>4. No intrahepatic bile duct stones or previous biliary surgery history | Once every 12 months | Once every 18 months |

**4.2 Follow-up Methods**

1. Routine Follow-up: Priority is given to the combination of "telephone follow-up + online questionnaire", conducted by trained follow-up specialists (nurses or research assistants). Each follow-up lasts 10–15 minutes, and the follow-up time and patient response status are recorded simultaneously.

2. On-site Follow-up: High-risk patients require at least 1 on-site follow-up (completed at the hepatobiliary surgery outpatient clinic) per year, while medium/low-risk patients require at least 1 on-site follow-up every 2 years. Physical examinations, laboratory tests, and imaging examinations are required during on-site follow-up.

3.Emergency Follow-up: If a patient reports suspected recurrence symptoms (e.g., abdominal pain, jaundice, fever ≥ 38.5℃) via phone, emergency follow-up should be initiated immediately. The patient is instructed to go to the hospital for treatment within 24 hours, and a green channel for outpatient service is coordinated simultaneously to avoid delayed diagnosis and treatment.

**5. Follow-up Content**

**5.1 Assessment of Clinical Symptoms and Signs**

1. Symptom Monitoring: A structured questionnaire is used to ask patients whether they have the following symptoms. If symptoms occur, the onset time, duration, inducing factors, and relief methods should be recorded:

Abdominal pain: Location (right upper abdomen/epigastric region), nature (colic/distending pain), severity (VAS score 0–10), and whether it radiates to the shoulder and back.

Jaundice: Onset time of skin/scleral yellowing, and whether it is accompanied by dark urine (tea-colored urine) or pale stool (clay-colored stool).

Infection-related symptoms: Fever (maximum body temperature, fever pattern), chills, nausea and vomiting, and loss of appetite.

2. Physical Examination: During on-site follow-up, focus on the following examinations:

- Abdominal signs: Tenderness and rebound tenderness in the right upper abdomen, Murphy’s sign, to determine the presence of biliary obstruction or infection signs.

- Systemic signs: Degree of skin and scleral yellowing, presence of skin pruritus, to evaluate the extent of liver function damage.

**5.2 Laboratory Index Testing**

Fasting venous blood is collected during each on-site follow-up and emergency visit to detect the following indicators. The results should be compared with the preoperative baseline values for analysis:

1. Liver function indicators: Direct bilirubin, alkaline phosphatase (ALP), gamma-glutamyl transferase (GGT), alanine transaminase (ALT), aspartate transaminase (AST) (reference ranges: direct bilirubin 3.4–17.1 μmol/L, ALP 40–150 U/L, GGT 7–45 U/L, ALT 5–40 U/L, AST 8–40 U/L).

2. Inflammatory indicators: White blood cell count (WBC) (4–10 × 10⁹/L), C-reactive protein (CRP) (0–10 mg/L), to evaluate the presence of biliary infection.

3.Coagulation function: Prothrombin time (PT) (11–13.5 seconds), activated partial thromboplastin time (APTT) (25–37 seconds), to indirectly reflect liver function reserve.

**5.3 Imaging Examinations**

1.Basic Examination: All patients undergo abdominal ultrasound during each follow-up (focusing on measuring the common bile duct diameter and detecting the presence of stone echoes). If abnormalities are indicated by ultrasound, further enhanced CT or MRCP is required.

2. Confirmatory Examination: If ultrasound/CT suggests stone recurrence, confirmation should be made via MRCP (to clarify the location, number, and maximum diameter of stones) or ERCP (which has both diagnostic and therapeutic functions). The stone clearance during ERCP should be recorded.

3. Adjustment of Examination Frequency: If a patient has abnormal laboratory indicators (e.g., direct bilirubin increases by > 50% compared with the baseline) or experiences suspected symptoms, imaging examinations should be conducted in advance without waiting for the regular follow-up time.

**5.4 Follow-up on Treatment and Lifestyle**

1.Postoperative Intervention Measures: Record whether the patient has received postoperative adjuvant treatment, such as oral ursodeoxycholic acid (UDCA) (dosage, course of treatment), T-tube indwelling time (if a T-tube was placed during surgery), and whether the patient has undergone endoscopic dilation for biliary stricture.

2. Lifestyle Factors:

Diet: Whether the patient has a long-term high-fat diet (≥ 3 times a week of fried/fatty foods), and daily water intake (daily water intake < 1500 ml is considered insufficient).

Bad Habits: Smoking (whether the patient currently smokes, daily smoking amount), drinking (frequency of drinking per week, amount of alcohol consumed each time).

Comorbidity Management: Medication adherence and indicator control of patients with hypertension, diabetes, or hyperlipidemia (e.g., whether blood pressure is < 140/90 mmHg, whether fasting blood glucose is < 7.0 mmol/L).

**6. Data Management and Quality Control**

1. Data Recording: An electronic data capture (EDC) system is used to record follow-up information. Data entry should be completed within 24 hours after each follow-up, and the entered content should be checked by two persons (each checked by a follow-up specialist and a research nurse) to ensure no omissions or errors.

2. Missing Data Handling: If a patient only completes part of the content during a follow-up (e.g., MRCP is not performed), the reason for the missing data (e.g., patient refusal, equipment failure) should be marked, and the missing items should be supplemented first during the next follow-up.

3. Quality Inspection: The research team conducts quality inspection on 10% of the follow-up records every month, including reviewing telephone recordings (to confirm the completeness of symptom inquiry) and checking the original inspection reports (to confirm the authenticity of imaging/laboratory results). The unqualified rate should be < 5%; otherwise, the follow-up specialists need to be retrained and the relevant records rechecked.

**7. Follow-up Ethics and Protection of Patients’ Rights and Interests**

1. Informed Consent: Patients must sign the *Informed Consent Form for Follow-up* before discharge, which clearly informs them of the purpose, content, frequency of follow-up, and privacy protection measures. The consent form should be filed in the patient’s medical record.

2. Privacy Protection: All follow-up data (e.g., name, ID number, inspection results) are stored in an encrypted manner, and only authorized researchers have access to the data. Disclosure to third parties is prohibited.

3. Medical Security: If stone recurrence or complications are found during follow-up, the patient should be immediately connected with the attending physician of hepatobiliary surgery to formulate a diagnosis and treatment plan. Hospitalization should be arranged if necessary to avoid delaying the patient’s condition due to follow-up.
