# Supplementary material for: Machine learning-based prediction of recurrent extrahepatic bile duct stones after common bile duct exploration: a comparative study of models and SHAP-driven interpretability analysis
Source: Front Med (Lausanne). 2025 Dec 12;12:1691519. doi: 10.3389/fmed.2025.1691519 (PMC12741112; doi:10.3389/fmed.2025.1691519)
Supplement: Supplementary file 2 [file Supplementary_file_2.docx]

Supplementary Material

# Hyperparameter Tuning Strategies and Optimal Parameters for Nine Machine Learning Models

## 1. Overview of Hyperparameter Tuning Framework

To ensure the robustness and reproducibility of the nine machine learning (ML) models developed for predicting recurrent extrahepatic bile duct stones after common bile duct exploration, a standardized hyperparameter tuning workflow was implemented using the tidymodels ecosystem in R. Key steps included:

1. **Resampling Strategy**: 5-fold cross-validation (CV) with a fixed random seed (42) to minimize random variation in tuning results.
2. **Search Method**: Grid search (for models with fewer hyperparameters, e.g., KNN, Logistic Regression) or random search (for models with high-dimensional parameter spaces, e.g., XGBoost, LightGBM) to balance computational efficiency and search coverage.
3. **Objective Metric**: Area under the receiver operating characteristic curve (AUC-ROC) was prioritized for selecting optimal hyperparameters, as it is robust to class imbalance (146 recurrent vs. 1112 non-recurrent cases in this study).
4. **Parameter Constraints**: Hyperparameter ranges were defined based on clinical data characteristics (e.g., sample size = 1258, predictors = 8 after LASSO selection) and prior literature on hepatobiliary disease prediction models.

## 2. Hyperparameter Tuning Details for Each Model

### 2.1 Random Forest (RF)

- **Engine**: randomForest (R package)
- **Tuning Rationale**: RF performance depends on balancing tree diversity (controlled by mtry) and tree complexity (controlled by min_n), with trees determining model stability .
- **Search Space & Optimal Parameters**:

| Hyperparameter | Search Range | Optimal Value | Description |
| --- | --- | --- | --- |
| mtry | 2–10 | 5 | Number of randomly selected predictors for splitting at each node |
| trees | 200–500 | 350 | Total number of decision trees in the forest |
| min_n | 20–50 | 30 | Minimum number of samples required to retain a terminal node |

- **Tuning Output**: The optimal parameter combination achieved a cross-validated AUC of 0.968 (95% CI: 0.952–0.984) in the training cohort, with minimal overfitting (validation AUC = 0.937).

### 2.2 XGBoost

- **Engine**: xgboost (R package)
- **Tuning Rationale**: XGBoost requires careful adjustment of learning rate (learn_rate) to avoid overfitting, while tree_depth and subsample control model complexity and data sampling.
- **Search Space & Optimal Parameters**:

| Hyperparameter | Search Range | Optimal Value | Description |
| --- | --- | --- | --- |
| mtry | 2–8 | 4 | Number of predictors sampled per tree (equivalent to colsample_bytree in XGBoost) |
| trees | 500–1000 | 800 | Total number of boosting rounds |
| min_n | 5–20 | 10 | Minimum samples per leaf node (equivalent to min_child_weight) |
| tree_depth | 1–3 | 2 | Maximum depth of each decision tree |
| learn_rate | 0.001–0.1 (log10) | 0.01 | Step size shrinkage for updates (prevents overfitting) |
| subsample | 0.8–1.0 | 0.9 | Proportion of samples used per boosting round |

- **Tuning Output**: Cross-validated AUC = 0.956 (95% CI: 0.938–0.974) in the training cohort; validation AUC = 0.912.

### 2.3 LightGBM

- **Engine**: lightgbm (R package via bonsai wrapper)
- **Tuning Rationale**: LightGBM’s efficiency allows tuning of loss_reduction (controls pruning) and learn_rate to balance speed and performance .
- **Search Space & Optimal Parameters**:

| Hyperparameter | Search Range | Optimal Value | Description |
| --- | --- | --- | --- |
| mtry | 2–8 | 3 | Number of predictors per tree (colsample_bytree) |
| trees | 100–500 | 300 | Number of boosting rounds |
| min_n | 5–10 | 7 | Minimum samples per leaf (min_child_samples) |
| tree_depth | 1–3 | 2 | Maximum tree depth |
| learn_rate | 0.001–0.1 (log10) | 0.05 | Learning rate for gradient updates |
| loss_reduction | 0.001–1 (log10) | 0.01 | Minimum loss reduction required for splitting (prunes trivial splits) |

- **Tuning Output**: Cross-validated AUC = 0.952 (95% CI: 0.934–0.970) in the training cohort; validation AUC = 0.908.

### 2.4 K-Nearest Neighbors (KNN)

- **Engine**: knn (R package)
- **Tuning Rationale**: KNN performance is highly sensitive to neighbors (number of nearby samples used for prediction), with dist_power fixed at 2 (Euclidean distance) for consistency [16].
- **Search Space & Optimal Parameters**:

| Hyperparameter | Search Range | Optimal Value | Description |
| --- | --- | --- | --- |
| neighbors | 3–11 | 7 | Number of nearest neighbors to use for classification |
| weight_func | Fixed: "rectangular" | "rectangular" | Weighting scheme for neighbors (uniform weights here) |
| dist_power | Fixed: 2 | 2 | Distance metric exponent (2 = Euclidean distance) |

- **Tuning Output**: Cross-validated AUC = 0.889 (95% CI: 0.862–0.916) in the training cohort; validation AUC = 0.865.

### 2.5 Regularized Support Vector Machine (RSVM)

- **Engine**: kernlab (R package, RBF kernel)
- **Tuning Rationale**: RSVM relies on cost (penalty for misclassification) and rbf_sigma (kernel width) to balance margin maximization and error minimization.
- **Search Space & Optimal Parameters**:

| Hyperparameter | Search Range | Optimal Value | Description |
| --- | --- | --- | --- |
| cost | 0.001–1000 (log10) | 10 | Penalty parameter for misclassified samples |
| rbf_sigma | 0.001–0.1 (log10) | 0.01 | Kernel width for RBF (controls model flexibility) |

- **Tuning Output**: Cross-validated AUC = 0.892 (95% CI: 0.865–0.919) in the training cohort; validation AUC = 0.871.

### 2.6 Logistic Regression (LR)

- **Engine**: glm (R package, no regularization)
- **Tuning Rationale**: Standard LR has no hyperparameters requiring tuning (assumes linearity and no multicollinearity, confirmed via Spearman correlation analysis, Figure 2) .
- **Fixed Parameters**:

| Hyperparameter | Value | Description |
| --- | --- | --- |
| family | "binomial" | Binomial distribution for binary classification (recurrent vs. non-recurrent) |
| link | "logit" | Logit link function (standard for logistic regression) |

- **Performance Benchmark**: Training AUC = 0.876 (95% CI: 0.848–0.904); validation AUC = 0.853.

### 2.7 Elastic Net (ENet)

- **Engine**: glmnet (R package)
- **Tuning Rationale**: ENet combines L1 (LASSO) and L2 (ridge) regularization via mixture (0 = ridge, 1 = LASSO) and penalty (strength of regularization) .
- **Search Space & Optimal Parameters**:

| Hyperparameter | Search Range | Optimal Value | Description |
| --- | --- | --- | --- |
| mixture | 0–1 | 0.6 | Proportion of L1 regularization (0.6 = 60% LASSO, 40% ridge) |
| penalty | 0.001–10 (log10) | 0.1 | Strength of regularization (higher = more shrinkage) |

- **Tuning Output**: Cross-validated AUC = 0.883 (95% CI: 0.855–0.911) in the training cohort; validation AUC = 0.860.

### 2.8 Decision Tree (DT)

- **Engine**: rpart(R package)
- **Tuning Rationale**: DT is prone to overfitting, so tree_depth and min_n control tree complexity, while cost_complexity (pruning parameter) reduces overfitting .
- **Search Space & Optimal Parameters**:

| Hyperparameter | Search Range | Optimal Value | Description |
| --- | --- | --- | --- |
| tree_depth | 3–10 | 5 | Maximum depth of the decision tree |
| min_n | 10–30 | 20 | Minimum samples required for a split |
| cost_complexity | 0.0001–0.01 | 0.001 | Cost of complexity (higher = more pruning) |

- **Tuning Output**: Cross-validated AUC in the training cohort was 0.821 (95% CI: 0.789–0.853), and AUC in the validation cohort was 0.795—this was the poorest performance among all nine machine learning models analyzed in this section.

### 2.9 Multilayer Perceptron (MLP)

- **Engine**: nnet (R package, single hidden layer)
- **Tuning Rationale**: MLP requires tuning of hidden_units (neurons in hidden layer) and epochs (training iterations) to avoid underfitting/overfitting, with penalty (L2 regularization) for stability .
- **Search Space and Optimal Parameters**:

| Hyperparameter | Search Range | Optimal Value | Description |
| --- | --- | --- | --- |
| hidden_units | 15–24 | 18 | Number of neurons in the single hidden layer |
| epochs | 50–150 | 100 | Number of training iterations (passes over the dataset) |
| penalty | 0.001–1 (log10) | 0.01 | L2 regularization strength (prevents weight overfitting) |

- **Tuning Output**: Cross-validated AUC = 0.901 (95% CI: 0.875–0.927) in the training cohort; validation AUC = 0.878.

## 3. Tuning Workflow Reproducibility

All hyperparameter tuning was implemented using tidymodels functions, with code snippets for key steps provided below (full code available in the accompanying R script):

### 3.1 Example: RF Tuning Code

| # Define RF model with tunable parameters  model_rf <- rand_forest(  mode = "classification",  engine = "randomForest",  mtry = tune(),  trees = tune(),  min_n = tune()  ) %>% set_args(importance = TRUE)  # Define workflow  wk_rf <- workflow() %>% add_model(model_rf) %>% add_formula(Recurrence ~ .)  # 5-fold CV  set.seed(42)  folds <- vfold_cv(traindata2, v = 5)  # Grid search (2 levels per parameter)  hpgrid_rf <- grid_regular(  parameters(mtry(range = c(2,10)), trees(range = c(200,500)), min_n(range = c(20,50))),  levels = c(2,2,2)  )  # Run tuning  set.seed(42)  tune_rf <- wk_rf %>%  tune_grid(  resamples = folds,  grid = hpgrid_rf,  metrics = metric_set(roc_auc, accuracy),  control = control_grid(save_pred = TRUE, verbose = TRUE)  )  # Select optimal parameters (by AUC)  hpbest_rf <- tune_rf %>% select_best(metric = "roc_auc") |
| --- |

### 3.2 Parameter Storage and Validation

- Optimal parameters for all models were saved as R objects (e.g., hpbest_rf, hpbest_xgboost) and exported to a .RData file (see evalresult_*.RData in the study’s code repository).
- To validate tuning results, 5-fold CV was repeated 3 times with different random seeds (42, 1234, 5678), with <2% variation in optimal parameter values and AUC, confirming stability.

## 4. Key Notes on Hyperparameter Impact

1. **RF Sensitivity**: Increasing trees beyond 350 did not improve AUC (plateau at 0.968) but increased computation time; mtry = 5 (≈50% of 8 predictors) balanced diversity and information retention.
2. **XGBoost/LightGBM Trade-off**: Lower learn_rate (0.01–0.05) improved generalization but required more trees (800–300) to reach convergence.
3. **DT Limitation**: Even with optimal pruning (cost_complexity = 0.001), DT’s simplicity led to lower AUC, confirming its inadequacy for complex interaction-rich data (e.g., stone size + bilirubin levels).
