# Supplementary material for: Machine learning-based prediction of recurrent extrahepatic bile duct stones after common bile duct exploration: a comparative study of models and SHAP-driven interpretability analysis
Source: Front Med (Lausanne). 2025 Dec 12;12:1691519. doi: 10.3389/fmed.2025.1691519 (PMC12741112; doi:10.3389/fmed.2025.1691519)
Supplement: Supplementary file 3 [file Supplementary_file_3.docx]

Supplementary Material

# **Variable Missing Data, Handling Methods, and Prediction Model Supplementary Reports**

## 1. Variable Missing Rate Statistics

To clarify the completeness of clinical data and avoid potential selection bias caused by excluding patients with incomplete medical records, we systematically counted the missing rates of key variables included in the initial analysis (n=1480patients before screening). The missing rate was calculated as the ratio of the number of patients with missing values for a specific variable to the total number of initially screened patients. Detailed results are presented in Table S1.

### Table S1. Missing Rates of Key Variables in Initially Screened Patients (n=1480)

| Variable Category | Variable Name | Number of Missing Cases | Missing Rate (%) |
| --- | --- | --- | --- |
| Demographics | Age | 0 | 0 |
|  | Gender | 0 | 0 |
|  | BMI | 12 | 0.81 |
| Laboratory Indicators | Direct Bilirubin | 28 | 1.9 |
|  | ALP (Alkaline Phosphatase) | 31 | 2.1 |
|  | GGT (Gamma-Glutamyl Transferase) | 25 | 1.7 |
|  | ALT (Alanine Aminotransferase) | 35 | 2.4 |
|  | AST (Aspartate Aminotransferase) | 33 | 2.2 |
|  | WBC (White Blood Cell Count) | 18 | 1.2 |
|  | CRP (C-Reactive Protein) | 21 | 1.4 |
|  | PT (Prothrombin Time) | 24 | 1.6 |
|  | APTT (Activated Partial Thromboplastin Time) | 27 | 1.8 |
| Imaging Findings | Maximum Stone Diameter | 42 | 2.8 |
|  | Common Bile Duct Diameter | 38 | 2.6 |
|  | Concurrent Intrahepatic Bile Duct Stones | 30 | 2 |
| Comorbidities & Surgery History | Hypertension | 15 | 1 |
|  | Diabetes Mellitus | 17 | 1.1 |
|  | Hyperlipidemia | 20 | 1.4 |
|  | Chronic Liver Disease | 22 | 1.5 |
|  | Previous Biliary Surgery | 14 | 0.9 |
|  | Intraoperative T-tube Placement | 9 | 0.6 |
| Clinical Symptoms | Pain | 16 | 1.1 |
|  | Fever | 19 | 1.38 |
| Follow-up Data | Follow-up Duration (≥3 months) | 53 | 3.85 |

As shown in Table S1, the missing rates of all variables were below 5%, with the highest missing rates observed for "Follow-up Duration (≥3 months)"and "Maximum Stone Diameter". Key predictors selected by LASSO regression had missing rates far below the 10% threshold that may introduce significant selection bias. This indicates high data completeness, and the exclusion of patients with incomplete medical records had minimal impact on the overall representativeness of the study population.

## 2. Missing Value Handling Methods

### 2.1 Pre-screening Strategy for Missing Data

Before formal data analysis, we conducted a systematic review of the medical records of all initially screened patients (n=1480). For variables with minor missingness (missing rate <5%), we adopted the following measures to maximize data retention:

1. **Laboratory and imaging variables**: For indicators such as Direct Bilirubin, ALP, Maximum Stone Diameter, and Common Bile Duct Diameter, we cross-checked electronic medical records with paper-based documents (e.g., laboratory test reports, imaging film interpretations) to supplement missing values.
2. **Comorbidity and surgery history variables**: For information on Hypertension, Diabetes Mellitus, and Previous Biliary Surgery, we consulted attending physicians and nursing records to confirm unclear or missing data.
3. **Follow-up data**: For missing Follow-up Duration information, we contacted patients or their family members via telephone to supplement records, reducing the number of patients excluded due to insufficient follow-up from 78 to 53.

### 2.2 Decision to Exclude Patients with Incomplete Records

After pre-screening and supplementary data collection, 117 patients still had incomplete key variables (e.g., simultaneous missing of Maximum Stone Diameter and Common Bile Duct Diameter, or missing Direct Bilirubin combined with Follow-up Duration <3 months). We decided to exclude these patients instead of using imputation methods, based on the following considerations:

1. **Low overall missing rate**: As shown in Table S1, all key variables had missing rates <5%, and the 117 excluded patients accounted for only 7.9% of the initial cohort—unlikely to significantly affect the study’s statistical power or population representativeness.
2. **Avoidance of imputation bias**: For clinically meaningful variables (e.g., Maximum Stone Diameter, Direct Bilirubin), imputation methods (e.g., mean imputation, multiple imputation) may introduce artificial errors. For example, imputing missing Maximum Stone Diameter with the cohort mean (9.66 mm) could obscure the actual recurrence risk difference between patients with large stones (>15 mm) and small stones (<10 mm)—a key finding of this study.
3. **Consistency with clinical practice**: In real-world clinical risk assessment, missing key indicators (e.g., imaging parameters for stone size) often prevents accurate recurrence risk evaluation. Excluding patients with incomplete key variables aligns with the clinical workflow of using complete data for risk stratification.

### 2.3 Baseline Characteristics Comparison Between Excluded and Included Patients

To verify whether excluding patients with incomplete records introduced selection bias, we compared the baseline characteristics of the excluded group (n=117) and the finally included group (n=1258), and calculated **Standardized Mean Differences (SMD)** (SMD < 0.1 indicates good baseline balance). Detailed results are presented in Table S2.

### Table S2. Baseline Characteristics Comparison Between Excluded and Included Patients

| Variable | Excluded Group (n=117) | Included Group (n=1363) | Standardized Mean Difference (SMD) | p-value |
| --- | --- | --- | --- | --- |
| Age, mean ± SD (years) | 57.2 ± 14.8 | 57.4 ± 14.9 | 0.01 | 0.892 |
| Gender, n (%) [Male] | 61 (52.1%) | 689 (50.5%) | 0.03 | 0.74 |
| BMI, mean ± SD (kg/m²) | 24.3 ± 3.5 | 24.2 ± 3.5 | 0.03 | 0.816 |
| Maximum Stone Diameter, mean ± SD (mm) | 9.7 ± 5.7 (n=98) | 9.6 ± 5.9 (n=1315) | 0.02 | 0.867 |
| Common Bile Duct Diameter, mean ± SD (mm) | 12.6 ± 5.8 (n=95) | 12.8 ± 5.8 (n=1325) | 0.03 | 0.791 |
| Direct Bilirubin, mean ± SD (μmol/L) | 27.1 ± 39.2 (n=92) | 26.7 ± 40.1 (n=1335) | 0.01 | 0.923 |
| Recurrence Rate,  n (%) [Yes] | 13 (11.1%) (n=89) | 156 (11.5%) (n=1363) | 0.02 | 0.875 |

*Note: For variables with partial missingness in the excluded group, only patients with available data were included in the calculation: n=98 for Maximum Stone Diameter, n=95 for Common Bile Duct Diameter, n=92 for Direct Bilirubin, and n=89 for Recurrence Rate.*

All SMD values were < 0.1, and all p-values > 0.05, confirming no statistically significant differences in baseline characteristics between the two groups. Excluding patients with incomplete records did not introduce selection bias.

## 3. TRIPOD Checklist Compliance

We adhered to the TRIPOD (Transparent Reporting of a Multivariable Prediction Model for Individual Prognosis or Diagnosis) statement to ensure transparent reporting of the prediction model. Compliance details are presented in Table S3.

### Table S3. TRIPOD Checklist Compliance

| Title | Compliance Details |
| --- | --- |
| Title and Abstract | The title and abstract clearly state the model’s purpose (predicting extrahepatic bile duct stone [EHBDS] recurrence after common bile duct exploration [CBDE]) and core methods (Random Forest [RF] model + SHAP analysis). |
| Background and Rationale | Explained the clinical burden of post-CBDE EHBDS recurrence and limitations of traditional risk assessment methods. |
| Objectives | Clearly defined three core objectives: model development, performance validation, and interpretability analysis of key predictors. |
| Study Design | Retrospective cohort study; specified a 7:3 split ratio for training (70%) and validation (30%) cohorts. |
| Participants | Defined inclusion/exclusion criteria, enrollment period (2010–2024), and data source (Huangshi Central Hospital and Honghu People's Hospital). |
| Outcome Measure | Defined "EHBDS recurrence" as radiologically confirmed reappearance of stones ≥3 months after CBDE; diagnosis was adjudicated by two senior radiologists. |
| Predictors | Listed 28 initial variables and 8 predictors selected via LASSO regression; provided detailed measurement methods for each variable. |
| Data Sources and Measurement | Specified data extraction from electronic medical records and paper-based archives; imaging data (e.g., MRCP, CT) were reviewed independently by two radiologists. |
| Sample Size | Final sample size (n=1258) met the "10 events per predictor" rule (recurrence events: 159; 159/8 ≈ 20 events per predictor). |
| Missing Data | Reported variable-specific missing rates (Table S1) and detailed handling methods (pre-screening, supplementary collection, exclusion); verified no bias via sensitivity analysis (Table S2). |
| Model Development | Described LASSO regression for variable selection, RF model training, and hyperparameter optimization (grid search + 5-fold cross-validation). |
| Model Specification | Provided key RF model parameters (e.g., number of decision trees = 500, maximum depth = 10) and output type (recurrence probability, range: 0.01–0.99). |
| Model Performance | Reported comprehensive performance metrics: AUC (training cohort: 97.99%, validation cohort: 93.66%), accuracy, sensitivity, specificity, calibration curves, and decision curve analysis (DCA) results. |
| Internal Validation | Used an independent validation cohort (30% of total samples) to evaluate performance; results confirmed consistent model robustness. |
| External Validation | Completed; an external cohort (n=105) was included to verify model generalization, with results reported in the main analysis of model performance. |
| Model Updates | Not applicable (no model updates during the study period). |
| Model Implementation | Developed a nomogram (Figure 8 in the main text) to visualize the model for clinical application. |
| Limitations | Discussed limitations:lack of long-term follow-up (>5 years), and potential ambiguity in distinguishing "residual stones" from "recurrent stones." |
| Interpretation | Used SHAP analysis to explain the contribution of key predictors (e.g., Maximum Stone Diameter >15 mm as a high-risk threshold) and their interactions. |
| Ethics Approval | Approved by the Institutional Ethics Committee of Huangshi Central Hospital (Approval No.: HCH-2024-012); informed consent was waived due to the retrospective nature of the study. |
| Data Sharing | De-identified data are available upon reasonable request to the corresponding author. |
| Funding | Stated no specific financial support for this study. |

## 4. PROBAST Bias Risk Assessment

We used the PROBAST (Prediction Model Risk of Bias Assessment Tool) to evaluate bias risk across six core domains. Results are presented in Table S4.

### Table S4. PROBAST Bias Risk Assessment (Low/High/Unclear)

| Domain | Risk Level | Rationale |
| --- | --- | --- |
| Participants | Low | Clear inclusion/exclusion criteria; baseline characteristics were balanced between excluded and included patients (Table S2), with no evidence of selection bias. |
| Predictors | Low | All predictors were measured via standard clinical procedures (e.g., laboratory tests, imaging); missing rates were <5% (Table S1), and no imputation was used to avoid artificial bias. |
| Outcome | Low | The outcome (EHBDS recurrence) was defined by objective radiological evidence (MRCP/ERCP) and adjudicated by two independent radiologists to minimize diagnostic bias. |
| Model Development | Low | LASSO regression was used to reduce overfitting; hyperparameters were optimized via 5-fold cross-validation; all performance metrics were reported transparently. |
| Model Validation | Low | An independent validation cohort (30% of total samples) was used; model performance in the validation cohort (AUC: 93.66%) was consistent with the training cohort (AUC: 97.99%), confirming robustness. |
| Model Reporting | Low | All key details were reported: variable missing rates, handling methods, model parameters, performance metrics, and limitations; a nomogram was provided for clinical application. |

**Overall Bias Risk**: Low (no high-risk domains identified; all domains met PROBAST criteria for low bias).

## 5. Model Card

To facilitate clinical application, we developed a "model card" summarizing key information about the RF-based EHBDS recurrence prediction model. Details are presented in Table S5.

### Table S5. Model Card for RF-Based Post-CBDE EHBDS Recurrence Prediction

| Item | Details |
| --- | --- |
| **Applicable Population** | Adult patients (age ≥18 years) diagnosed with extrahepatic bile duct stones (EHBDS) who underwent common bile duct exploration (CBDE) as the primary surgical treatment, with a follow-up duration of ≥3 months. |
| **Input Variables** | 8 key predictors (all measurable via routine clinical tests):1. Age (years)2. Maximum Stone Diameter (mm)3. Common Bile Duct Diameter (mm)4. Concurrent Intrahepatic Bile Duct Stones (Yes/No)5. Previous Biliary Surgery (Yes/No)6. Direct Bilirubin (μmol/L)7. ALP (U/L)8. GGT (U/L) |
| **Output Result** | 1. Probability of post-CBDE EHBDS recurrence (range: 0.01–0.99)2. Risk stratification:- Low risk: Recurrence probability ≤0.2 - Moderate risk: Recurrence probability 0.2–0.6 - High risk: Recurrence probability >0.6 |
| **Failure Modes** | 1. Inapplicable to patients treated with ERCP (Endoscopic Retrograde Cholangiopancreatography) or EST (Endoscopic Sphincterotomy) (not CBDE).2. Reduced accuracy in patients with rare chronic liver diseases (e.g., primary biliary cholangitis) not included in the original cohort.3. Unreliable results if ≥2 input variables are missing (no imputation supported). |
| **Performance Metrics** | Validation cohort performance: - AUC: 93.66% - Accuracy: 0.902 - Sensitivity: 0.813 - Specificity: 0.915 |
| **Clinical Application** | 1. Guide follow-up frequency: High-risk patients (probability >0.6): follow-up every 3 months; Moderate-risk patients (0.2–0.6): follow-up every 6 months; Low-risk patients (≤0.2): follow-up every 12 months.2. Support targeted interventions: Consider prophylactic ursodeoxycholic acid treatment for high-risk patients to reduce recurrence risk. |
